# PCSK9 inhibitors for lipid-lowering treatment in patients with atherosclerotic cardiovascular disease: a health technology assessment

**DOI:** 10.3389/fcvm.2026.1853767

**Published:** 2026-08-07

**Authors:** Jingjing Lan, Yulan Qiu, Shuaijia Wang, Ying Zhang, Shiqi Cheng, Siying Chen, Yuanming Xing

**Affiliations:** 1 Department of Pharmacy, The First Affiliated Hospital of Xi'an Jiaotong University, Xi'an, Shaanxi, China; 2 Department of Cardiovascular Medicine, The First Affiliated Hospital of Xi'an Jiaotong University, Xi'an, Shaanxi, China

**Keywords:** atherosclerotic cardiovascular disease, health technology assessment, markov model, meta-analysis, PCSK9 inhibitors

## Abstract

**Background:**

In multiple countries, clinical guidelines recommend proprotein convertase subtilisin/kexin type 9 (PCSK9) inhibitors for lowering low-density lipoprotein cholesterol (LDL-C) in individuals with atherosclerotic cardiovascular disease (ASCVD). Comprehensive comparisons of the efficacy, safety, and cost-effectiveness across different PCSK9 inhibitors remain limited, and current evidence requires updating. To address this gap, this study applies a health technology assessment framework to systematically evaluate the use of PCSK9 inhibitors in patients with ASCVD.

**Methods:**

A systematic search of PubMed, EMBASE, and the Cochrane Library was conducted from database inception to February 28, 2026 to identify randomized controlled trials (RCTs) comparing PCSK9 inhibitors with standard care (statins with or without ezetimibe) in patients with ASCVD. Both pairwise and network meta-analyses were applied to synthesize direct and indirect evidence. Trial sequential analysis (TSA) was performed to evaluate the sufficiency of the accumulated data. In addition, a Markov model was developed from the perspective of the Chinese healthcare system to assess the cost-effectiveness of PCSK9 inhibitors in ASCVD patients.

**Results:**

In total, 24 RCTs comprising 63,328 participants were included. The results of TSA and network meta-analysis showed that, compared with SOC, all three PCSK9 inhibitors significantly reduced LDL-C levels. No statistically significant differences were found for stroke outcomes. However, both alirocumab and evolocumab significantly reduced the risk of non-fatal myocardial infarction. None of the three PCSK9 inhibitors was associated with an increased risk of serious adverse events. Using the meta-analysis findings and data from the Chinese healthcare system, the incremental cost-effectiveness ratios (ICERs) for alirocumab, evolocumab, and inclisiran versus standard care were estimated at USD 12,791.07/QALY, USD 11,646.38/QALY, and USD 31,730.14/QALY, respectively. In a scenario analysis incorporating a 72.1% reduction in the price of inclisiran, its ICER decreased to USD 9,686.31/QALY. The one-way sensitivity analysis showed that the discount rate exerted the greatest influence on model outcomes. In the price-reduction scenario, probabilistic sensitivity analysis indicated that inclisiran had a 96.6% probability of being cost-effective at a willingness-to-pay threshold of USD 13,410/QALY.

**Conclusion:**

Currently, evolocumab demonstrates relative advantages in lipid-lowering efficacy, cardiovascular outcomes, and economic value for patients with ASCVD. Alirocumab represented a cost-effective alternative. In the scenario analysis incorporating a 72.1% reduction in the price of inclisiran, inclisiran became a potentially highly cost-effective option in the Chinese healthcare setting.

## 1 Introduction

Atherosclerotic cardiovascular disease (ASCVD) continues to be a major cause of mortality globally, contributing to about 31% of all deaths (1). Its high rates of disability and recurrence not only substantially impair patients' quality of life but also impose a considerable disease and economic burden on both individuals and society (2, 3). In current approaches to ASCVD prevention and treatment, low-density lipoprotein cholesterol (LDL-C) is well recognized as a crucial modifiable risk factor, and intensive lipid-lowering therapy can markedly decrease the incidence of cardiovascular events (4–6). Although clinical presentations vary widely across patients with stable ASCVD, acute coronary syndrome (ACS), myocardial infarction, ischemic stroke, post-percutaneous coronary intervention (PCI) status, and diabetes-associated ASCVD, these conditions share a common pathophysiological basis of atherosclerosis and are managed using similar secondary prevention strategies aimed at reducing residual cardiovascular risk through LDL-C lowering. However, in real-world clinical settings, over half of patients at high or very high risk of ASCVD do not reach the LDL-C targets recommended by clinical guidelines (7, 8).

Proprotein convertase subtilisin/kexin type 9 (PCSK9) inhibitors reduce circulating LDL-C levels by blocking PCSK9 activity, which increases the availability of low-density lipoprotein receptors (LDLRs) on hepatocytes (9). In recent years, several randomized controlled trials (RCTs) have shown that adding PCSK9 inhibitors to statin therapy, as an intensified lipid-lowering approach, allows a higher proportion of patients with ASCVD to reach LDL-C targets and provides significant cardiovascular benefits. As a result, PCSK9 inhibitors have emerged as one of the most robustly supported non-statin lipid-lowering therapies (10–15). Consequently, they have been progressively incorporated into both international and domestic clinical guidelines and are recommended for secondary prevention in patients with high-risk or very high-risk ASCVD (4–6).

From a mechanistic perspective, different strategies targeting PCSK9 may influence the stability of long-term efficacy and real-world treatment management. Monoclonal antibody PCSK9 inhibitors, such as alirocumab and evolocumab, primarily exert their effects by blocking the interaction between circulating PCSK9 and LDLRs, and are characterized by a rapid onset of action and well-established lipid-lowering efficacy. In contrast, small interfering RNA agents, exemplified by inclisiran, achieve more sustained target inhibition by suppressing PCSK9 synthesis within hepatocytes, and their lower dosing frequency may improve patient adherence during long-term therapy (16). These mechanistic and pharmacological differences may not be fully captured in RCTs or meta-analyses that predominantly focus on biochemical endpoints. However, in the context of long-term chronic disease management and health economic evaluation, such differences may translate into variations in treatment persistence, dosing-related healthcare resource utilization, and cost-effectiveness outcomes, thereby influencing the overall value assessment of PCSK9-targeted treatment strategies.

Despite the well-established lipid-lowering efficacy and cardiovascular benefits of PCSK9 inhibitors, their high acquisition costs and limited accessibility have been major barriers to widespread clinical adoption. In recent years, China has incorporated selected PCSK9 inhibitors into the medical insurance reimbursement system through the National Drug Price Negotiation (NPDN) policy, substantially reducing patients' financial burden and facilitating their use across healthcare institutions at different levels (2). However, existing evidence suggests that, despite improved accessibility, the long-term economic sustainability of PCSK9 inhibitors in real-world payment settings remains highly dependent on drug pricing, target populations, and the magnitude of therapeutic benefit. To date, a systematic and comprehensive value assessment within the Chinese healthcare context is still lacking (17).

Given the growing burden of ASCVD, persistently low rates of LDL-C target attainment, and the restricted use of PCSK9 inhibitors because of their high cost, this study conducted a systematic evaluation of the efficacy, safety, and cost-effectiveness of three widely used PCSK9 inhibitors in patients with ASCVD within a health technology assessment (HTA) framework. The aim was to generate evidence to support informed and rational decision-making regarding the use of these agents in Chinese patients with ASCVD.

## 2 Methods

### 2.1 Meta-analysis

The meta-analysis and cost-effectiveness evaluation were performed and reported in line with the Preferred Reporting Items for Systematic Reviews and Meta-Analyses guidelines and the 2022 Consolidated Health Economic Evaluation Reporting Standards checklist (Supplementary Tables S1 and S2).

#### 2.1.1 Selection criteria

We included all RCTs on lipid-lowering therapy and prevention of cardiovascular events in patients with ASCVD. Studies were eligible if they met the following criteria: (1) RCT; (2) participants were adults (≥18 years) with ASCVD; (3) the intervention group received PCSK9 inhibitors, while the control group was treated with standard treatment (statins ± ezetimibe); and (4) the study reported changes in LDL-C, ApoB, or Lp(a) from baseline; along with at least one clinical outcome, including stroke, non-fatal myocardial infarction (NMI), coronary revascularization, or serious adverse events (SAE).

Studies were excluded if they met any of the following criteria: (1) the study population is not entirely composed of ASCVD patients; (2) the findings were unpublished or the necessary data were not accessible; (3) duplicate reports.

#### 2.1.2 Search strategy

The literature search covered PubMed, EMBASE, and the Cochrane Central Register of Controlled Trials. RCTs published from database inception to February 28, 2026 were eligible for inclusion. The search strategy incorporated both Medical Subject Headings terms and free-text keywords, such as “ASCVD,” “cardiovascular,” “coronary artery,” “PCSK9 inhibitor,” “alirocumab,” “evolocumab,” and “inclisiran.” Detailed search strategies are presented in Supplementary Material 1. Additionally, the reference lists of all included studies and relevant reviews were manually screened to identify any further eligible RCTs.

#### 2.1.3 Data extraction and quality assessment

Two investigators (LJ and WS) independently conducted data extraction and assessed study quality. Any disagreements were resolved through discussion, and a third investigator (XY) was consulted when necessary to achieve consensus.

The extracted information included the first author, publication year, and baseline characteristics (sample size, age, sex, and initial lipid profile), as well as details on treatment regimens and dosages, follow-up duration, and efficacy and safety outcomes. The primary endpoint was the change in LDL-C. Secondary endpoints comprised the incidence of SAEs, stroke, and NMI. SAEs were defined as treatment-emergent if they occurred after the initiation of study therapy or within 7 days after the final dose.

The methodological quality of the included RCTs was assessed using the Cochrane Risk of Bias tool implemented in Review Manager version 5.4 (Cochrane Collaboration).

#### 2.1.4 Statistical analysis

Three analytical approaches were applied: pairwise meta-analysis, trial sequential analysis (TSA), and network meta-analysis (NMA). For LDL-C, the direction of effect in the network meta-analysis was defined such that positive standardized mean differences (SMDs) indicate greater LDL-C reduction compared with standard of care. Accordingly, larger positive SMD values represent greater lipid-lowering efficacy. Sensitivity analysis was used to evaluate the impact of the quality of included studies and population heterogeneity on the outcomes. Detailed descriptions of the statistical methods used for the meta-analysis are provided in Supplementary Appendix 2.

The transitivity assumption was assessed by comparing potential effect modifiers across studies, including patient age, baseline LDL-C level, ASCVD subtype, background statin intensity, ezetimibe use, follow-up duration, and outcome definitions. Although clinical heterogeneity was present, the included studies were considered sufficiently comparable with respect to the therapeutic indication and mechanism of PCSK9-targeted treatment to support indirect comparisons. Because the network contained no closed loops among the three PCSK9 inhibitors, formal loop-specific inconsistency testing could not be performed. Network geometry was therefore examined descriptively, and sensitivity analyses excluding lower-quality studies and selected high-risk populations were performed to assess the robustness of the findings.

### 2.2 Cost-effectiveness analysis

#### 2.2.1 Model structure overview

A Markov model with a 20-year time horizon, which approximates the remaining lifetime of the modeled ASCVD cohort, was developed from the perspective of the Chinese healthcare system. Using TreeAge Pro 2021 (TreeAge Software, Inc., MA, USA), the long-term occurrence and progression of cardiovascular events in patients with ASCVD were simulated to assess the cost-effectiveness of different PCSK9 inhibitor treatment strategies.

In the economic evaluation, to enhance the contextual relevance and interpretability of the results, standard of care (SOC) was specifically defined as atorvastatin combined with ezetimibe. In the included clinical trials, background standard of care generally consisted of maximally tolerated statin therapy with or without ezetimibe. For the economic evaluation, a single standardized SOC regimen (atorvastatin plus ezetimibe) was selected to represent contemporary routine lipid-lowering therapy in the Chinese healthcare system. This approach was adopted to provide a consistent comparator across all treatment strategies and to avoid introducing additional variability arising from heterogeneous background lipid-lowering regimens (5). The intervention strategies were defined as alirocumab (75 mg every 2 weeks), evolocumab (140 mg every 2 weeks), or inclisiran (300 mg on day 1, day 90, and every 6 months thereafter), each administered in combination with SOC.

Based on the constructed Markov model, the long-term survival and disease progression of patients with ASCVD were projected (Supplementary Figure 1). The model comprised six health states: stable ASCVD, non-fatal stroke, post-stroke maintenance therapy, NMI, post–myocardial infarction maintenance therapy, and death (including cardiovascular-related and non-cardiovascular death). Consistent with previously published models, revascularization was incorporated into the model as a one-time medical event in the cost calculations rather than as an independent health state (3, 18). All transition processes in the model were adjusted using a half-cycle correction.

The model employed an annual cycle (cycle length = 1 year) to simulate patient outcomes over a 20-year time horizon. Taking into account the average life expectancy in China and the baseline age of the included ASCVD population, a maximum simulation duration of 20 years was set to reflect long-term disease progression and survival outcomes in a real-world context.

#### 2.2.2 Model inputs

##### 2.2.2.1 Intervention effects

The interventional effect parameters of each PCSK9 inhibitor on LDL-C reduction and cardiovascular clinical outcomes in the model were derived from the meta-analysis results. The treatment effects of PCSK9 inhibitors used in the economic model were estimated as the incremental effects relative to the background SOC reported in the included trials. Because PCSK9 inhibitors were administered as add-on therapy in all eligible studies, the relative treatment effects were assumed to be transferable to the standardized SOC regimen adopted in the model. Due to the absence of randomized controlled trial data on cardiovascular outcomes for inclisiran, it was not possible to directly estimate the effects of all three PCSK9 inhibitors on cardiovascular and all-cause mortality within a single analysis. Consequently, an indirect method based on the extent of LDL-C reduction was used to derive treatment effects for these endpoints (19, 20) (see Supplementary Appendix 3 for details). The interventional effect parameters for each treatment strategy are presented in Supplementary Table S6.

##### 2.2.2.2 Transition probability

The model incorporated transition probabilities reflecting the risks of NMI, stroke, and mortality in patients with ASCVD. Baseline event incidences were derived from age-specific all-cause mortality, cardiovascular and cerebrovascular mortality, and myocardial infarction rates reported in the China Health Statistics Yearbook^1^, and were adjusted for age in 5-year increments.

##### 2.2.2.3 Cost

All original cost inputs were collected in Chinese yuan (CNY) and converted to US dollars (USD) using the average 2025 exchange rate of 1 USD = 7.14 CNY. All cost results, including total costs, incremental costs, and ICERs, are reported in USD in the revised manuscript. In line with the Chinese Pharmacoeconomic Evaluation Guidelines, both costs and health utilities were discounted at an annual rate of 5%. Sensitivity analyses were performed by varying the discount rate between 0% and 8%.

##### 2.2.2.4 Utility

Utility values for each health state were obtained from previously published studies involving Chinese or other Asian populations, covering conditions such as stable ASCVD, NMI, and stroke (Supplementary Table S3).

The modeled cohort was based on the pooled baseline characteristics of the included ASCVD population, with a median age of 61.3 years. Transition probabilities for myocardial infarction, stroke, cardiovascular death, and non-cardiovascular death were derived from the meta-analysis and age-specific Chinese epidemiological data. Health-state utility values were obtained from published studies involving Chinese or other Asian populations, while direct medical costs were estimated from the perspective of the Chinese healthcare system. All costs were converted to 2025 US dollars and both costs and health outcomes were discounted at an annual rate of 5%. Detailed information on the model inputs used in the cost-effectiveness analysis is provided in Supplementary Appendix 3.

#### 2.2.3 Cost-effectiveness analysis

The analysis estimated total costs, quality-adjusted life years (QALYs), and incremental cost-effectiveness ratios (ICERs) for patients with ASCVD across different treatment strategies. ICERs were calculated as the ratio of additional costs to additional QALYs gained between two interventions2. In accordance with Chinese pharmacoeconomic evaluation guidelines, the willingness-to-pay (WTP) threshold was set at USD 13,410–40,230, equivalent to one to three times the national per capita gross domestic product (GDP) in 2024. Cost-effectiveness was categorized as follows: ICERs below 1 × GDP/QALY were considered highly cost-effective; those between 1 and 3×GDP/QALY were regarded as cost-effective; and values exceeding 3×GDP/QALY were classified as not cost-effective.

To evaluate the robustness of the model, both one-way sensitivity analysis and probabilistic sensitivity analysis (PSA) were performed. In the one-way sensitivity analysis, each parameter was varied independently while all other inputs were held constant, allowing assessment of its effect on the net monetary benefit (NMB) and the overall sensitivity of the model outcomes to key variables. Given a specified WTP threshold, NMB was defined as the product of incremental QALYs and the WTP minus the incremental cost. The range of parameter variation was preferentially set as the 95% CI; for parameters without available variance information, a ± 20% variation around the baseline value was assumed. The findings of the one-way sensitivity analysis are illustrated using tornado diagrams.

PSA was performed using a Monte Carlo simulation with 1,000 iterations to assess how simultaneous variation in model inputs influences overall uncertainty in the results. Appropriate probability distributions were assigned to different parameter types: utility values were modeled using beta distributions, cost parameters using gamma distributions, and the relative risks of PCSK9 inhibitors combined with SOC versus SOC alone, including NMI, non-fatal stroke, all-cause mortality, and cardiovascular mortality—were modeled using log-normal distributions (Supplementary Table S3). The PSA results are presented as cost-effectiveness acceptability curves (CEACs) and incremental cost-effectiveness scatter plots.

The model structure, health states, transition pathways, calculations, and key assumptions were independently checked for internal consistency. The robustness of the model was further evaluated through deterministic sensitivity analysis, probabilistic sensitivity analysis, and scenario analyses.

#### 2.2.4 Scenario analysis

To further evaluate the impact of lowering LDL-C on cardiovascular mortality, we conducted a scenario analysis based on the latest evidence-based studies (21, 22). This included cost-effectiveness analyses based on the assumptions of a 10% and 20% reduction in cardiovascular mortality for every 1 mmol/L decrease in LDL-C, as well as assessments over two time horizons: 10 years and 25 years.

## 3 Results

### 3.1 Meta-analysis

#### 3.1.1 Characteristics of included trials

**Figure 1:**
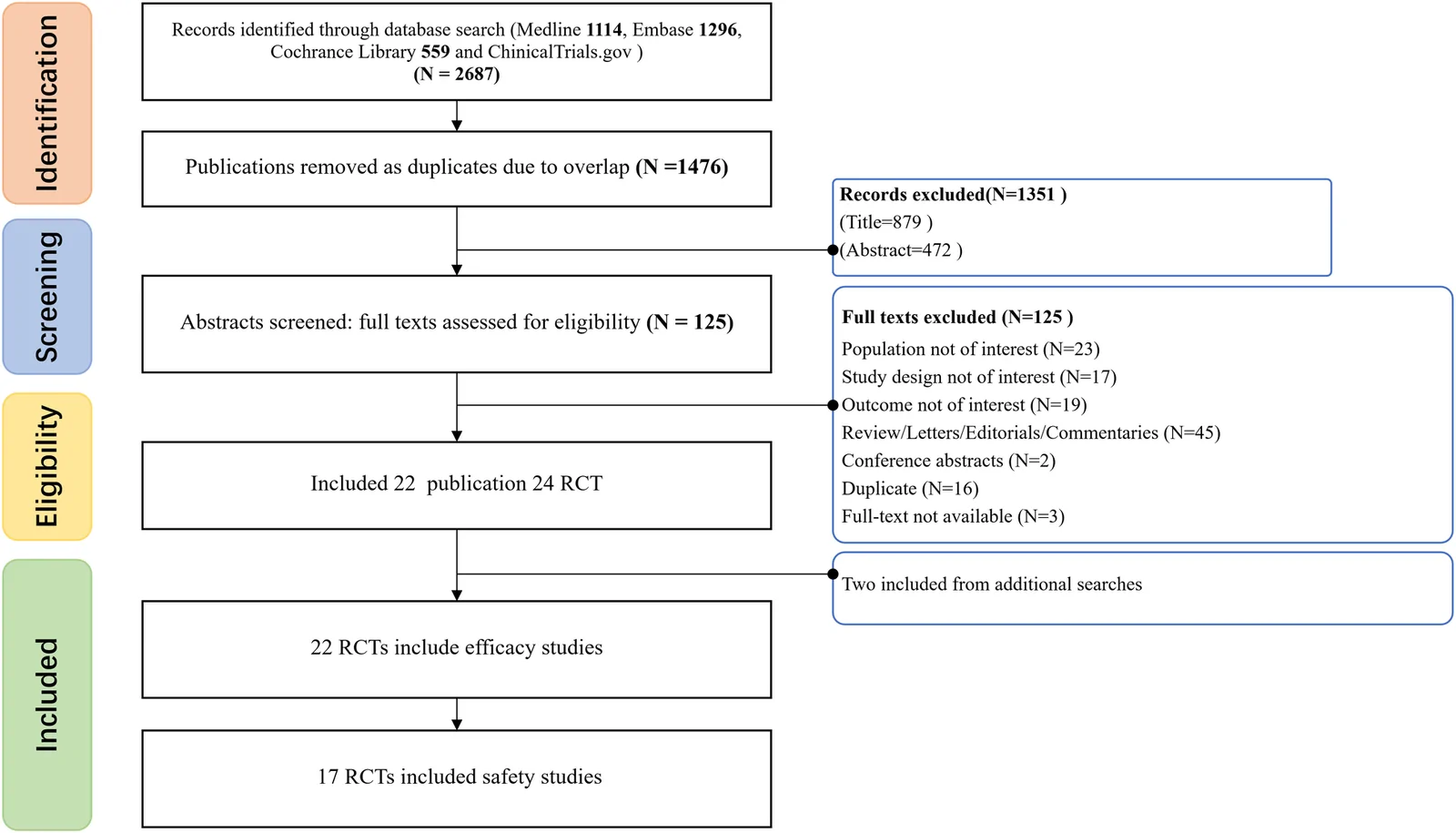
Flow diagram of the literature selection process for evaluating the efficacy and safety of PCSK9 inhibitors in patients with ASCVD. PCSK9, proprotein convertase subtilisin/kexin type 9; ASCVD, atherosclerotic cardiovascular disease.

**Figure 2:**
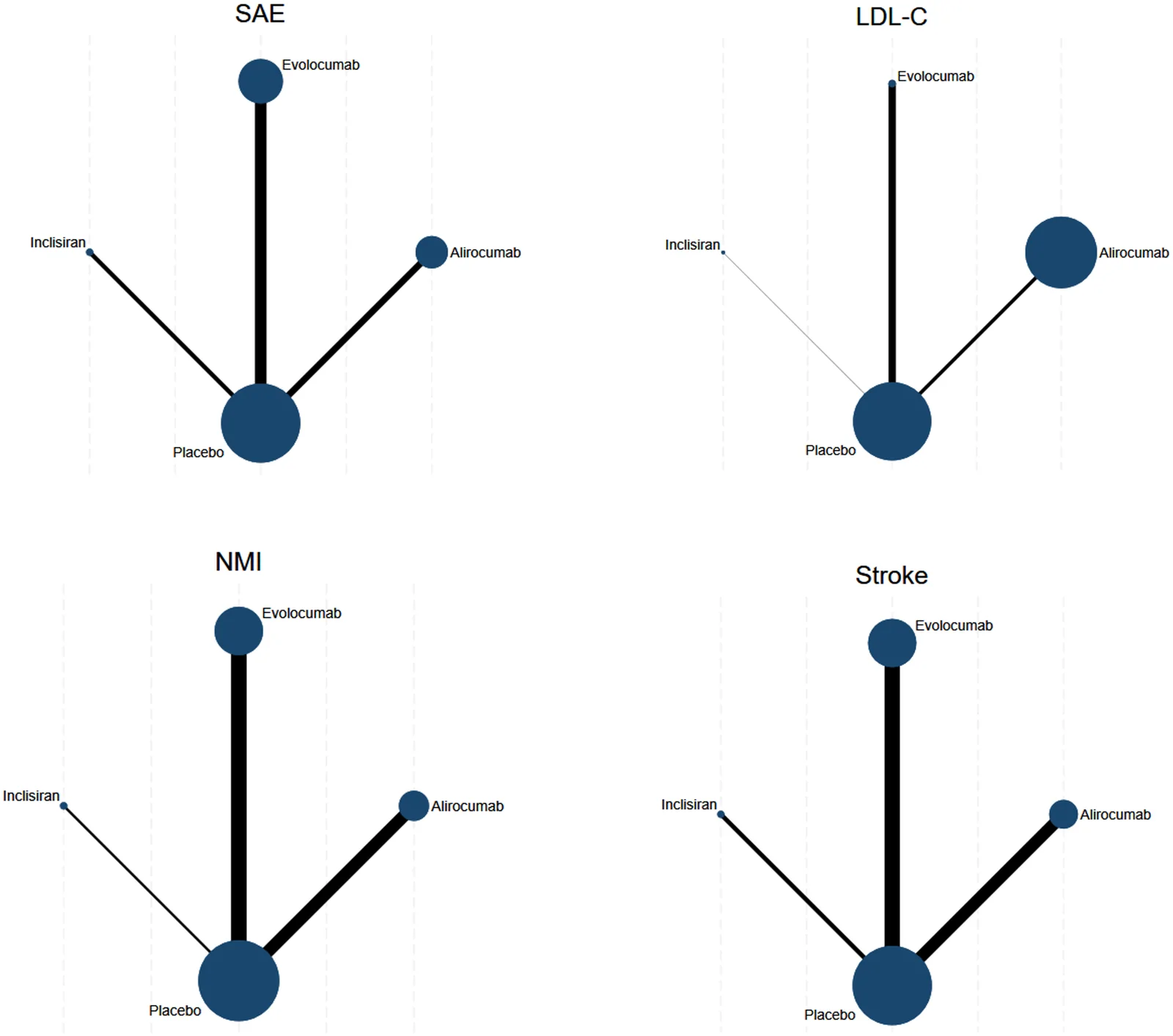
Network plots of eligible comparisons for all outcomes. Node size reflects the number of studies included. Interventions that were directly compared are connected by lines, with line thickness proportional to the number of studies evaluating each comparison. As there are no direct head-to-head comparison studies among the three PCSK9 inhibitors, the network structures in the figure are all open-loop. LDL-C, low-density lipoprotein cholesterol; NMI, non-fatal myocardial infarction; SAE, serious adverse event.

An initial search identified 1,476 records published from database inception to February 28, 2026. After title and abstract screening, 1,351 articles were excluded, and the remaining 125 articles were assessed through full-text review (Figure 1). Ultimately, 24 RCTs were included, assessing the efficacy and safety of alirocumab (8 trials), evolocumab (12 trials), and inclisiran (4 trials) in comparison with control treatments. The risk of bias for the included trials is illustrated in Supplementary Figures S1 and S2. A total of 63,328 participants were analyzed, with a median age of 61.3 years. Key characteristics of the studies are summarized in Table 1, and the network structures for the primary outcomes are presented in Figure 2.

**Table 1:** Key characteristics of studies included in the meta-analysis.

Study No.	Study	Use of PCSK9 inhibitors	Sample size	Median age	Sex(male/female)	Patient characteristics	Follow-up time	Baseline LDL-C concentrations(mmol/L)	Changes from baseline LDL-C (mmol/L)	High-dose statin use at screening	Ezetimibe use	Proportion of patients with diabetes	Prior myocardial infarction or stroke
1	Dean J. Kereiakes, et al. (23)	Alirocumab	209	63	131/78	Clinical ASCVD or subclinical ASCVD	52w	2.45	−1.18	129/209	15/209	94/209	206/209
2	Christopher P. Cannon, et al. (24)	Alirocumab	479	61.7	360/119	Equivalent risks of CHD or CHD	52w	2.8	−1.42	320/479	-	147/479	477/479
3	Jennifer G. Robinson, et al. (25)	Alirocumab	1,553	60.4	983/570	Clinical ASCVD or subclinical ASCVD	78w	3.17	−1.92	727/1,553	216/1,553	1055/1,553	542/1,553
4	G.G. Schwartz, et al. (10)	Alirocumab	9,462	58.5	7,072/2,390	ACS	2.8y	2.38	−1.33	8,380/9,462	269/9,462	2,693/9,462	2,096/9,462
5	Lorenz Räber, et al. (12)	Alirocumab	148	58.4	124/24	AMI	52w	4	−3.39	11/148	0/148	16/148	2/148
6	Stephen J Nicholls, et al.1 (13)	Evolocumab	484	59.8	349/135	CAD	76w	2.39	−1.45	280/484	9/484	98/484	169/484
7	Marc S Sabatine, et al. (14)	Evolocumab	13,784	62.5	10,397/3,387	ASCVD	2.2y	2.37		9,585/13,784	726/13,784	5,054/13,784	13,784/13,784
8	Stephen J Nicholls, et al.2 (26)	Evolocumab	80	60.9	60/20	NSTEMI	52w	3.63	−2.95	63/80	1/80	13/80	5/80
9	Kausik K Ray, et al.1 (15)	Inclisiran	781	66.4	535/246	ASCVD	540d	2.7		525/781	80/781	371/781	781/781
10	Kausik K Ray, et al.2 (15)	Inclisiran	810	64.8	579/231	Clinical ASCVD or subclinical ASCVD	540d	2.77		640/810	52/810	296/810	712/810
11	B Cariou, et al.1 (27)	Alirocumab	51	54.9	29/22	ASCVD (Type 1 Diabetes)	24w	3.3		15/51	2/51	51/51	11/51
12	B Cariou, et al.2 (27)	Alirocumab	294	63.9	161/133	ASCVD (Type 2 Diabetes)	24w	2.8		87/294	45/294	294/294	119/294
13	Yan Hao, et al (28)	Evolocumab	68	62.2	45/23	Diagnosed with ACS and underwent PCI	12w	3.54	−2.96	-	-	27/68	65/68
14	Shamir R Mehta,et al (29)	Alirocumab	38	61.4	27/11	Patients undergoing PPCI for STEMI	12w	2.97	−2.22	-	2/38	5/38	4/38
15	Michael A Vavuranakis, et al (30)	Evolocumab	74	56.9	29/45	NMI	30d	2.33	−1.44	34/39	4/39	18/39	19/39
16	Michelle L O'Donoghue, et al (31)	Evolocumab	3,355	62.4	2,582/773	ASCVD	5y	2.37		2,584/3,355	200/3,355	1,121/3,355	2,807/3,355
17	Liang Wang, et al (32)	Evolocumab/ Alirocumab	36	63.9	28/8	ACS	24w	3.04	−1.78	-	-	-	-
18	Wen Tian, et al. (33)	Evolocumab	136	63.6	100/36	Acute ischemic stroke	1y	2.76		-	-	58/136	48/136
19	Yusuke Akiyama, et al. (34)	Evolocumab	23	62.3	19/4	Patients who undergo coronary artery stenting	28w	2.14	−1.63	-	7/23	9/23	11/23
20	Hiroki Uehara, et al. (35)	Evolocumab	29	59.6	22/7	ACS	36w	2.63	−1.78	-	-	-	-
21	Xiaohui Qiu, et al. (36)	Evolocumab	60	61	46/14	Acute ischemic stroke	8w	3.15	−2.32	-	-	21/60	4/60
22	Yong Huo, et al. (37)	Inclisiran	170	58.8	130/40	ASCVD/ASCVD equivalent risk	330d	2.81		118/170	-	71/170	163/170
23	Yanyan Han, et al. (38)	Evolocumab	22	44.4	18/4	Premature coronary artery disease	52w	2.85	−2.02	-	-	2/22	-
24	Michael J Koren, et al. (11)	Inclisiran	225	66	158/67	ASCVD	330d	2.51	−1.5	-	1/225	95/225	211/225

ASCVD, atherosclerotic cardiovascular disease; ACS, acute coronary syndrome; AMI, acute myocardial infarction; CAD, coronary artery disease; CHD, coronary heart disease; NMI, non-fatal myocardial infarction; NSTEMI, non-ST-segment elevation myocardial infarction; PCI, percutaneous coronary intervention; PCSK9, proprotein convertase subtilisin/kexin type 9; SAE, serious adverse event.

### 3.2 Pairwise meta-analysis

#### 3.2.1 Primary outcome

**Figure 3:**
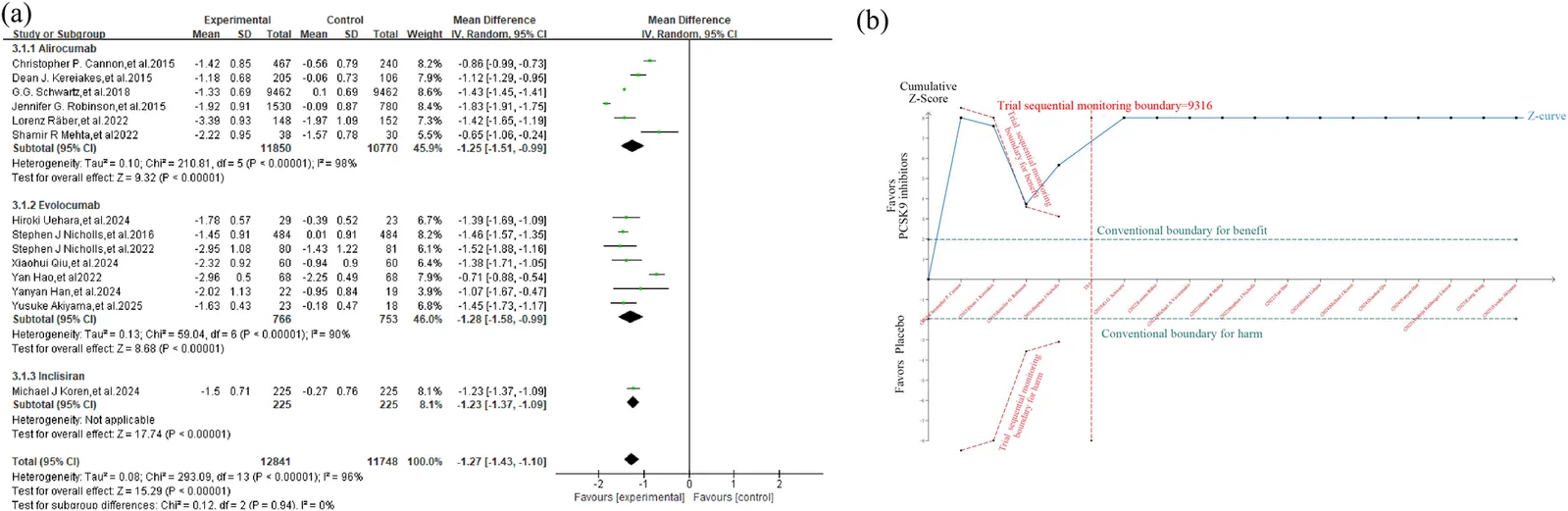
Effects of PCSK9 inhibitors compared to standard of care on LDL-C. **(A)** Forest plot of the pairwise meta-analysis of LDL-C reduction; **(B)** trial sequential analysis of LDL-C reduction. PCSK9, proprotein convertase subtilisin/kexin type 9; CI, confidence interval.

Fourteen RCTs evaluated LDL-C reduction by comparing PCSK9 inhibitors with SOC. The findings showed that treatment with PCSK9 inhibitors led to a significant decrease in LDL-C levels (MD, −1.27; 95% CI, −1.43 to −1.10; *P* < 0.01; Figure 3A).

In addition, TSA indicated that the cumulative Z-curve crossed both the conventional and trial sequential monitoring boundaries for benefit and entered the benefit region. This suggests that the available evidence is sufficient and conclusive, and that further trials are unlikely to be required (Figure 3B). The funnel plot (Supplementary Figure S3) displayed an approximately symmetrical distribution of points around the central line, indicating no apparent publication bias. Subgroup analyses for the three PCSK9 inhibitors are summarized in Supplementary Table S4, with all agents demonstrating significant reductions in LDL-C levels.

#### 3.2.2 Secondary outcomes

Sixteen RCTs reported the incidence of SAEs, comparing PCSK9 inhibitors with SOC. The findings indicated that the incidence of SAEs did not differ significantly between the PCSK9 inhibitor group and SOC (RR, 0.98; 95% CI, 0.95–1.01; *P* = 0.14; Supplementary Figure S5a). In addition, TSA showed that the cumulative Z-curve did not cross the conventional or trial sequential monitoring benefit boundaries but reached the required information size. This indicates that the available evidence is sufficient and conclusive, and that further trials are unlikely to be needed (Supplementary Figure S5b). The funnel plot (Supplementary Figure S6) showed that the scatter points were approximately symmetrically distributed on both sides of the midline, suggesting no evidence of publication bias.

In addition, treatment with PCSK9 inhibitors was linked to a significant decrease in Lp(a) levels (MD, −22.83; 95% CI, −26.05 to −19.61; *P* < 0.01; Supplementary Figure S7), apoB levels (MD, −36.80; 95% CI, −47.13 to −26.47; *P* < 0.01; Supplementary Figure S8), the incidence of NMI (RR, 0.80; 95% CI, 0.74–0.86; *P* < 0.01; Supplementary Figure S9), and stroke (RR, 0.76; 95% CI, 0.66–0.87; *P* < 0.01; Supplementary Figure S10). The funnel plot (Supplementary Figure S11) showed that the scatter points were approximately symmetrically distributed on both sides of the midline, suggesting no evidence of publication bias.

Sensitivity analyses based on study quality and patient population (Supplementary Figures S11–S18) demonstrated consistent results across all four outcomes (LDL-C, SAEs, NMI, and stroke), indicating that the pooled estimates were robust and not materially influenced by between-study heterogeneity or inclusion of high-risk subpopulations.

### 3.3 Network meta-analysis

#### 3.3.1 Primary outcome

Fourteen RCTs assessed the impact of PCSK9 inhibitors on LDL-C levels. NMA showed that, compared with SOC, alirocumab, evolocumab, and inclisiran all produced significant reductions in LDL-C (SMD 0.28, 95% CI: 0.21–0.37; 0.31, 95% CI: 0.24–0.40; and 0.29, 95% CI: 0.14–0.60, respectively). In the network meta-analysis, the direction of effect was defined such that positive SMD values indicate greater LDL-C reduction relative to SOC; therefore, larger positive SMD values represent greater lipid-lowering efficacy. No statistically significant differences in LDL-C–lowering efficacy were observed among the different PCSK9 inhibitors.

**Figure 4:**
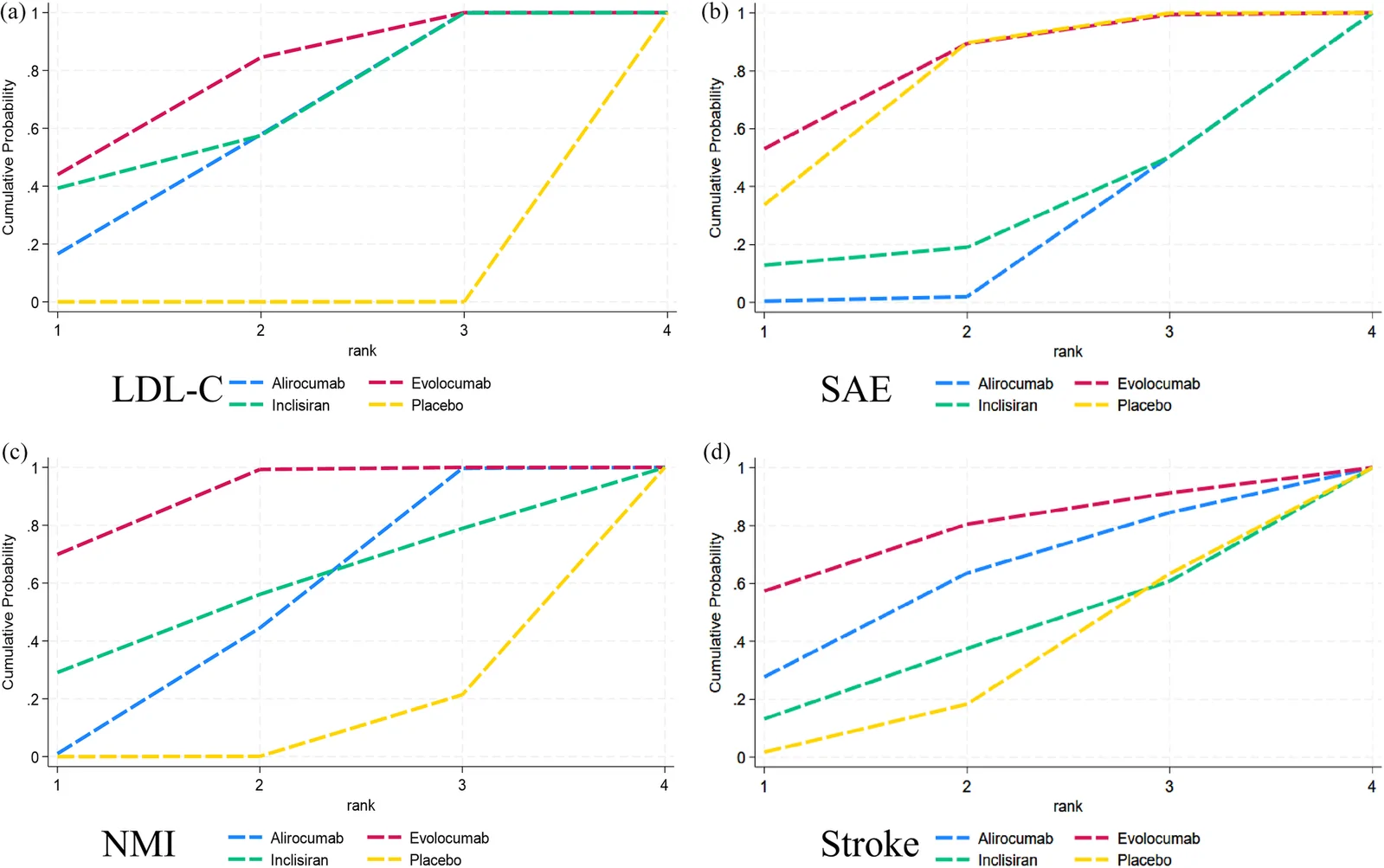
SUCRA diagram of efficacy and safety. The blue dashed line represents alirocumab, the red dashed line represents evolocumab, the green dashed line represents inclisiran, and the yellow dashed line represents the standard of care group. SUCRA, surface under the cumulative ranking curve; LDL-C, Low-density lipoprotein cholesterol; SAE, Serious adverse event; NMI, Non-fatal myocardial infarction.

To describe the relative ranking of each PCSK9 inhibitor, we calculated the SUCRA probabilities (Supplementary Table S5) and plotted the corresponding cumulative ranking plots. According to the SUCRA analysis, evolocumab (SUCRA = 0.75) had the highest probability of achieving a favorable ranking for LDL-C reduction, followed by inclisiran (SUCRA = 0.67) and alirocumab (SUCRA = 0.577) (Figure 4A).

#### 3.3.2 Secondary outcomes

Sixteen RCTs were included in the analysis of SAEs. NMA indicated that, compared with SOC, alirocumab was associated with a lower risk of SAEs (RR = 0.93, 95% CI: 0.87–0.99). The SUCRA analysis showed that evolocumab (SUCRA = 0.806) and placebo (SUCRA = 0.744) had similar rankings for SAE outcomes, indicating comparable risks of SAEs. In contrast, alirocumab (SUCRA = 0.176) and inclisiran (SUCRA = 0.274) had lower SUCRA values, suggesting lower rankings for SAE outcomes, that is, a relatively lower probability of SAEs (Figure 4B, Supplementary Table S5). However, because SAEs comprised heterogeneous events reported across different trials and were not necessarily treatment-related, this finding should be interpreted cautiously.

Sixteen RCTs were eligible for the analysis of NMI. Compared with SOC, both evolocumab (RR = 0.73, 95% CI: 0.65–0.81) and alirocumab (RR = 0.86, 95% CI: 0.77–0.96) significantly reduced the risk of NMI. The SUCRA analysis indicated that the effectiveness in preventing NMI ranked highest for evolocumab, followed by inclisiran, and alirocumab (Figure 4C, Supplementary Table S5).

Fourteen RCTs assessed the effectiveness of PCSK9 inhibitors in preventing stroke. In the NMA, none of the agents showed a statistically significant reduction in stroke risk compared with SOC, although a general trend toward decreased risk was observed. Based on the SUCRA probabilities, the ranking for stroke prevention was evolocumab, followed by alirocumab, and inclisiran (Figure 4D, Supplementary Table S5).

### 3.4 Cost-effectiveness analysis

#### 3.4.1 Base-case analysis

In the base-case analysis, treatment with SOC incurred the lowest total cost, with an average total cost over the 20-year horizon of USD 1,625.57 and an effectiveness of 1.25 QALYs. As shown in Table 2, the average total cost over the 20-year horizon of alirocumab, evolocumab, and inclisiran were USD 3,672.14, USD 3,605.46, and USD 6,702.39, respectively. Compared with SOC, the corresponding incremental costs were USD 2,046.57, USD 1,979.89, and USD 5,076.82. Based on the unrounded model outputs, the ICERs of alirocumab, evolocumab, and inclisiran versus SOC were USD 12,791.07/QALY, USD 11,646.38/QALY, and USD 31,730.14/QALY, respectively. In the scenario incorporating a 72.1% reduction in the acquisition price of inclisiran, its average total cost over the 20-year horizon decreased to USD 3,175.38, corresponding to an incremental cost of USD 1,549.81 versus SOC. Based on the unrounded model outputs, the ICER decreased to USD 9,686.31/QALY.

**Table 2:** Cost-effectiveness results for PCSK9 inhibitors in patients with atherosclerotic cardiovascular disease: base-case and scenario analyses.

Group	Total cost (USD)	QALYs	Incremental cost (USD)	Incremental QALYs	Incremental cost-utility ratio (USD/QALY)
Base-case analysis					
SOC	1,625.57	1.25			
alirocumab	3,672.14	1.41	2,046.57	0.16	**12,791.07**
evolocumab	3,605.46	1.42	1,979.89	0.17	**11,646.38**
inclisiran	6,702.39	1.41	5,076.82	0.16	31,730.14
72.1% reduction in the price of inclisiran					
inclisiran	3,175.38	1.41	1,549.81	0.16	**9,686.31**
Scenario analysis					
For every 1 mmol/L decrease in LDL-C, cardiovascular mortality decreases by 20%.					
SOC	1,625.57	1.25			
alirocumab	3,736.86	1.44	2,111.28	0.19	**11,112.02**
evolocumab	3,668.77	1.44	2,043.2	0.19	**10,753.69**
inclisiran	6,827.21	1.44	5,201.64	0.19	27,377.05
72.1% reduction in the price of inclisiran					
inclisiran	3,230.05	1.44	1,604.47	0.19	**8,444.60**
For every 1 mmol/L decrease in LDL-C, cardiovascular mortality decreases by 10%.					
SOC	1,625.57	1.25			
alirocumab	3,552.63	1.36	1,927.06	0.11	17,518.69
evolocumab	3,417.73	1.36	1,792.16	0.11	16,292.32
inclisiran	3,151.86	1.35	1,526.29	0.1	15,262.86
Simulate the situation over a 10-year period.					
SOC	1,038.8	0.67			
alirocumab	2,071.99	0.77	1,033.18	0.1	**10,331.85**
evolocumab	2,033.67	0.77	994.86	0.1	**9,948.64**
inclisiran	1,803.85	0.76	765.05	0.09	**8,500.54**
Simulate the situation over a 25-year period.					
SOC	1,622.77	1.63			
alirocumab	4,130.47	1.8	2,507.7	0.17	14,751.19
evolocumab	4,020.17	1.81	2,397.4	0.18	**13,318.9**
inclisiran	3,506.34	1.8	1,883.57	0.17	**11,079.85**

QALY, quality-adjusted life year; LDL-C, low-density lipoprotein cholesterol; SOC, standard of care. All costs are reported in 2025 US dollars using an exchange rate of 1 USD=7.14 CNY. Incremental costs, incremental QALYs, and ICERs were calculated relative to SOC. The ICER of USD 9,686.31/QALY for inclisiran was obtained from the price-reduction scenario and does not represent the base-case result.

Bold values indicate ICERs below the willingness-to-pay threshold of USD 13,410/QALY (one time the Chinese per capita GDP), representing highly cost-effective treatment strategies.

#### 3.4.2 Sensitivity analysis

**Figure 5:**
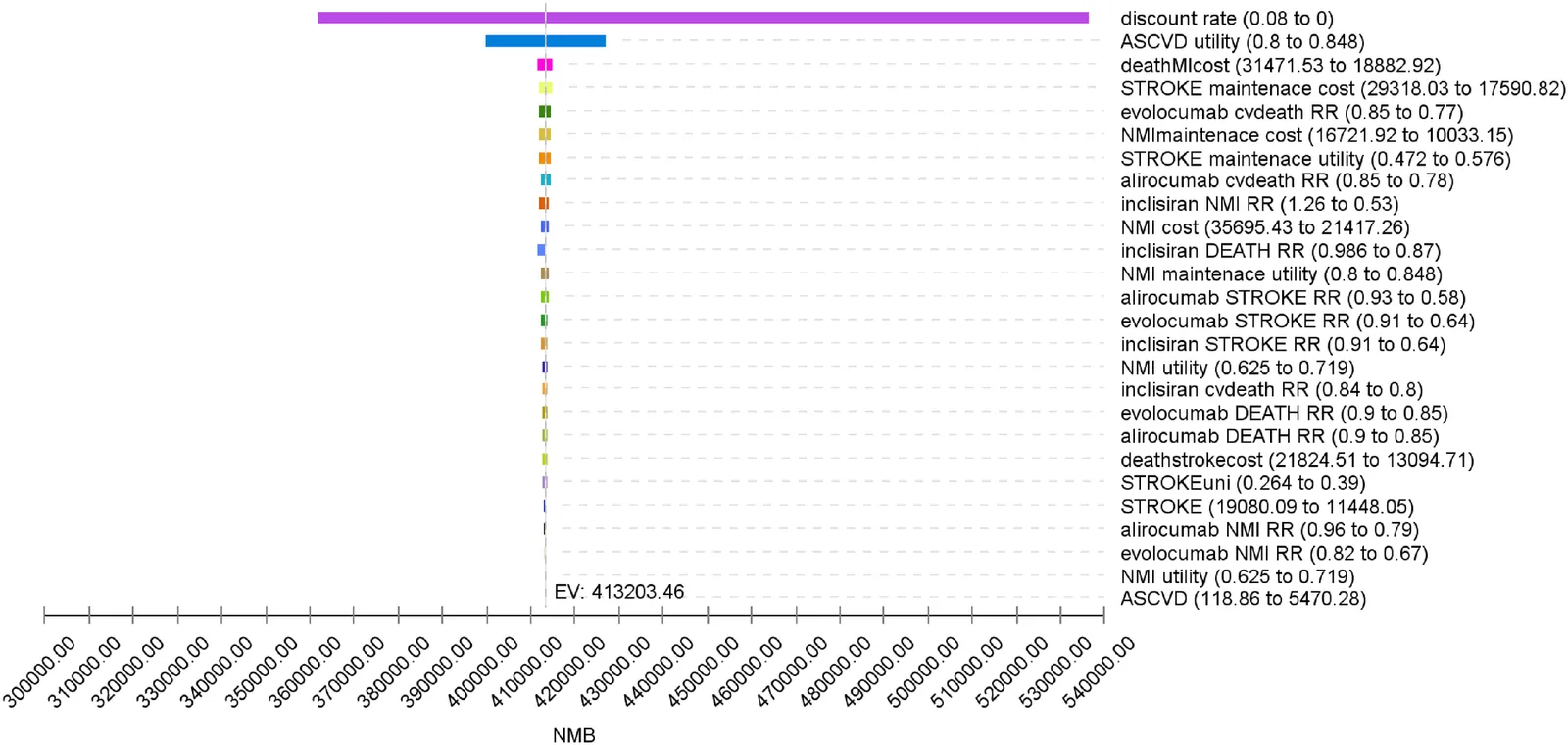
Tornado plot illustrating the results of the deterministic sensitivity analysis.

One-way sensitivity analysis demonstrated that the base-case results were robust to changes in individual parameters (Figure 5). The most influential factors included the discount rate, the utility value for patients with ASCVD, the cost of death due to myocardial infarction, the cost of stroke maintenance treatment, and the cardiovascular mortality rate in the evolocumab group. Despite their impact, variations in these parameters did not change the overall conclusions of the cost-effectiveness analysis.

**Figure 6:**
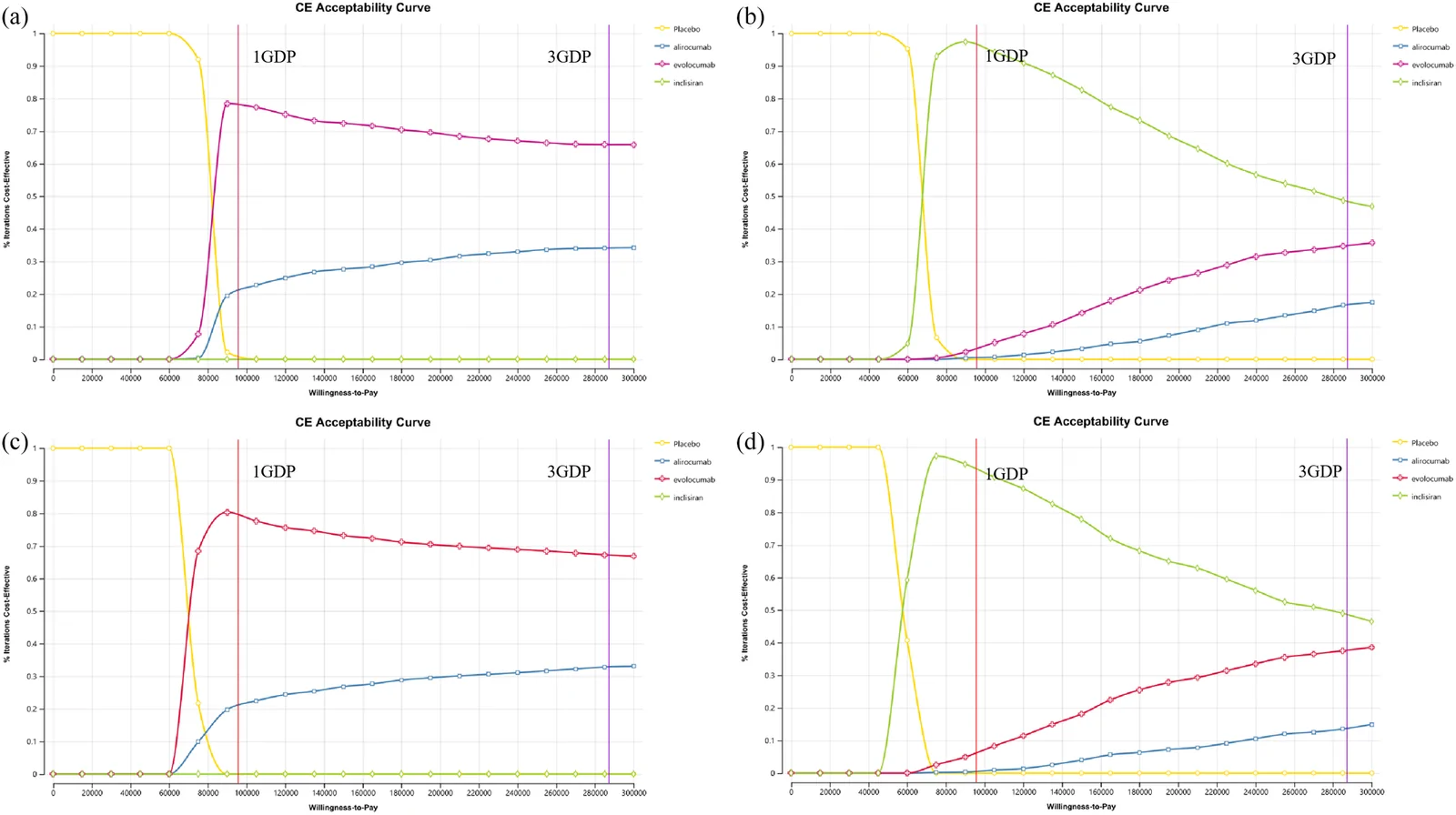
Cost-effectiveness acceptability curves of PCSK9 inhibitors and standard of care. **(a)** Base-case analysis; **(b)** base-case scenario with a 72.1% reduction in inclisiran price; **(c)** a scenario assuming a 20% decrease in cardiovascular mortality for each 1 mmol/L reduction in LDL-C; and **(d)** a combined scenario incorporating both a 72.1% price reduction for inclisiran and a 20% decrease in cardiovascular mortality per 1 mmol/L LDL-C reduction. The blue curve indicates the probability of alirocumab being cost-effective as WTP increases, while the pink curve represents evolocumab, the green curve represents inclisiran, and the gold curve corresponds to placebo. The red vertical line denotes WTP = 1  ×  GDP, and the purple vertical line represents WTP = 3 × GDP. WTP, willingness-to-pay; GDP, gross domestic product; LDL-C, low-density lipoprotein cholesterol.

The CEAC derived from the probabilistic sensitivity analysis is presented in Figure 6A. At a WTP threshold of USD 13,410/QALY, evolocumab was the most cost-effective treatment option, with a probability of 79%, followed by alirocumab with a probability of 21%. At a WTP threshold of USD 40,230/QALY, both evolocumab and alirocumab were more cost-effective than standard care, with evolocumab showing a 65.9% probability of being the most cost-effective option and alirocumab 34.1%. In contrast, inclisiran had a 0% probability of being the optimal strategy at any WTP threshold, indicating that it was not cost-effective under the current analysis.

**Figure 7:**
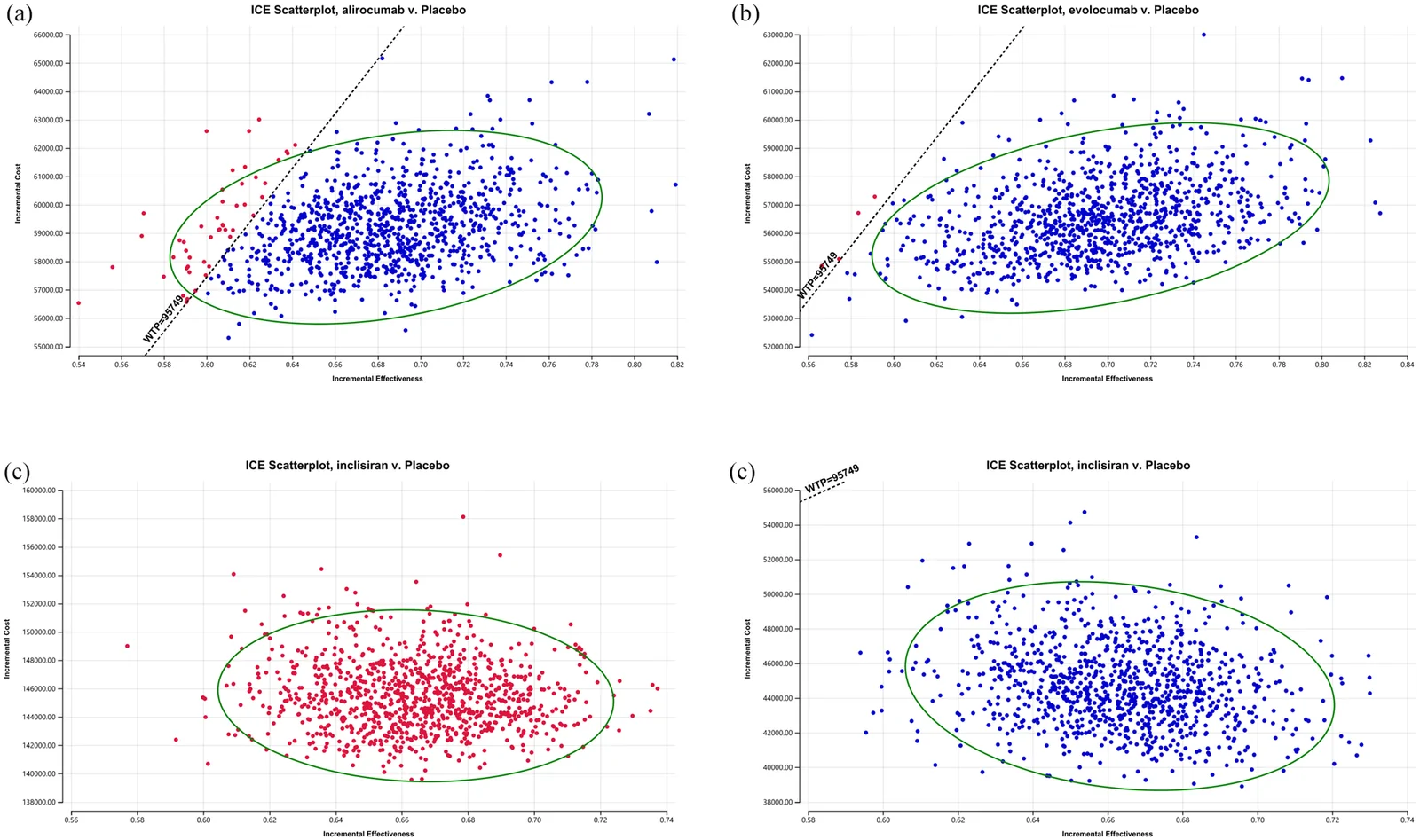
Scatter plot of incremental cost-effectiveness of PCSK9 inhibitors compared with SOCs. **(a)** Base-case analysis, alirocumab vs SOC; **(b)** base-case analysis, evolocumab vs SOC; **(c)** base-case analysis, inclisiran vs SOC; **(d)** base-case analysis with a 72.1% price reduction for inclisiran, inclisiran vs SOC; SOC, standard of care; LDL-C, low-density lipoprotein cholesterol.

Incremental cost-effectiveness scatter plots were used to compare the likelihood of PCSK9 inhibitors versus SOC being cost-effective across different WTP thresholds (Figure 7). The results showed that at a WTP of USD 13,410, the probabilities of being cost-effective compared with SOC were 95.1% for alirocumab and 99.6% for evolocumab, whereas inclisiran was not cost-effective (Figures 7A–C).

#### 3.4.3 Scenario analysis

In an additional price-reduction scenario, the acquisition cost of inclisiran was reduced by 72.1%, while all other model parameters remained unchanged. Under this scenario, the total cost over the 20-year horizon of the inclisiran strategy decreased to USD 3,175.38, and its ICER versus SOC decreased from USD 31,730.14/QALY in the base-case analysis to USD 9,686.31/QALY. This result should be interpreted as a scenario-specific estimate rather than as the base-case finding. At a WTP threshold of USD 13,410/QALY, inclisiran had a 96.6% probability of being cost-effective in this price-reduction scenario.

Under the scenario assuming a 10% reduction in cardiovascular mortality for each 1 mmol/L decrease in LDL-C, the ICERs for all three PCSK9 inhibitors were within the range of 1–3×GDP per capita. Under the scenario assuming a 20% reduction in cardiovascular mortality per 1 mmol/L LDL-C decrease, the ICERs for all three agents decreased substantially, demonstrating improved cost-effectiveness.

In the time horizon sensitivity analysis, compared with the 20-year base-case scenario, the ICERs of evolocumab and inclisiran increased in the 10-year scenario, although both remained highly cost-effective, while alirocumab remained within the 1–3×GDP per capita range. In the 25-year scenario, ICERs for all three drugs also increased compared with the 20-year base-case scenario; evolocumab and inclisiran remained highly cost-effective, whereas alirocumab remained within the 1–3×GDP per capita range.

Overall, all scenario analyses were consistent with the direction of the base-case results. ICER values decreased with increasing assumed cardiovascular mortality benefit. The 20-year time horizon yielded the lowest ICERs and the most favorable cost-effectiveness results. When the time horizon was shortened (10 years) or extended (25 years), ICERs increased, indicating that the model results were moderately sensitive to assumptions regarding the time horizon.

## 4 Discussion

Because traditional statins used for lipid-lowering therapy are associated with adverse effects and suboptimal adherence, and studies have shown that the LDL-C–lowering benefits achieved with statins are not irreplaceable (39), statins nevertheless remain the cornerstone of lipid-lowering therapy. However, in routine clinical practice, many patients fail to reach guideline-recommended LDL-C targets despite receiving maximally tolerated statin therapy, while others are unable to sustain long-term treatment due to statin intolerance.

These challenges have led to growing interest in non-statin lipid-lowering therapies for patients with ASCVD. Ezetimibe lowers LDL-C by inhibiting intestinal cholesterol absorption and provides additional LDL-C reduction when combined with statins. Clinical studies have also demonstrated its cardiovascular benefits, leading to its early inclusion in guideline recommendations as a non-statin option (40). The introduction of PCSK9 inhibitors has further transformed the landscape of lipid-lowering therapy. At present, in multiple countries, adding PCSK9 inhibitors to SOC is recommended as a lipid-lowering strategy for patients with ASCVD, and the findings of our study further support the effectiveness of this approach.

To date, few RCTs or high-quality observational studies have directly compared different PCSK9 inhibitors combined with SOC in patients with ASCVD, limiting the ability to perform head-to-head, evidence-based comparisons. Therefore, the use of NMA is necessary to indirectly evaluate the comparative efficacy and safety of different PCSK9 inhibitors. Previous studies have explored this topic only partially. Wang et al. (41) investigated the efficacy and safety of PCSK9 inhibitors in secondary prevention populations, focusing primarily on all-cause mortality, but did not examine differences in LDL-C reduction. Although our findings are generally consistent with theirs, our study included a larger number of RCTs, applied TSA to demonstrate the robustness of the evidence, and additionally evaluated LDL-C changes and cost-effectiveness. Similarly, a recent analysis by Raone et al. (42). examined clinical outcomes in patients with established ASCVD, focusing on major adverse cardiovascular events, but did not incorporate LDL-C changes. Moreover, the limited availability of cardiovascular outcome data for inclisiran may introduce bias into the results of such analyses. In addition, existing pharmacoeconomic studies have predominantly focused on comparisons between individual PCSK9 inhibitors and SOC, and comprehensive cost–benefit comparisons of different PCSK9 inhibitors within a unified analytical framework in ASCVD patients are still lacking. This gap is particularly relevant in the Chinese context, where inclisiran has recently been included in the national medical insurance reimbursement system and its economic value warrants further evaluation (43).

Elevated LDL-C levels are strongly linked to a higher risk of cardiovascular events and increased healthcare costs. Previous studies have systematically compared the efficacy and safety of PCSK9 inhibitors, statins, and ezetimibe in patients with hyperlipidemia, consistently showing that PCSK9 inhibitors achieve greater reductions in LDL-C and cardiovascular events while maintaining an overall favorable safety profile (44, 45). The meta-analysis in the present study confirms that PCSK9 inhibitors significantly lower LDL-C in patients with ASCVD, and TSA demonstrates that the cumulative evidence has reached the prespecified information size, indicating high stability and reliability. It should be noted that SUCRA-based rankings reflect the relative probability of being ranked among treatments and should not be interpreted as evidence of statistically significant differences between interventions. Among the evaluated agents, evolocumab showed relatively higher LDL-C–lowering efficacy in ranking analysis, consistent with prior findings (44). In the network meta-analysis, alirocumab was associated with a lower incidence of SAEs compared with SOC. However, SAEs comprised heterogeneous clinical events across the included trials and were not necessarily attributable to the study treatment. Therefore, this finding should be interpreted as an observed trial-level association rather than definitive evidence of a superior safety profile (41).

For NMI, evolocumab ranked first in the SUCRA analysis. Although inclisiran also ranked relatively high, its risk ratio did not reach statistical significance, which may be attributable to the limited availability of cardiovascular data and the relatively small number of events reported for inclisiran (15). Consequently, the current evidence based on indirect comparisons more robustly supports the benefits of evolocumab and alirocumab in the prevention of NMI (41), whereas the clinical outcome benefits of inclisiran still require confirmation in further RCTs. For stroke outcomes, pairwise meta-analyses indicated that alirocumab and evolocumab were linked to a lower risk of stroke, while the NMA did not show statistically significant differences. This inconsistency may be explained by the overall low incidence of stroke events, limited direct comparative evidence, and the resulting increased uncertainty in effect estimates. Although the SUCRA rankings continue to suggest a relative advantage of evolocumab and alirocumab in stroke prevention, their clinical benefits should be further validated by additional studies with stroke as a primary endpoint.

This study further evaluated the long-term economic value of PCSK9 inhibitors for patients with ASCVD from the perspective of the Chinese healthcare system. Economic evaluation is of critical importance in addressing resource constraints within healthcare systems. Although the model adopted a 20-year time horizon rather than a true lifetime simulation, this duration closely approximates the remaining life expectancy of the modeled cohort (median baseline age 61.3 years) in the Chinese population and has been widely adopted in previous pharmacoeconomic evaluations. Using cardiovascular outcome data from the long-term FOURIER-OLE trial (46), the base-case assumptions included a 15% reduction in cardiovascular mortality and a 10% reduction in all-cause mortality for each 1 mmol/L decrease in LDL-C. Additionally, the effects of PCSK9 inhibitors on cardiovascular and all-cause mortality were obtained from published literature.

Recent studies have shown that the evolocumab-based regimen is the most cost-effective option for patients with ASCVD in China (43, 46) and also demonstrates the greatest economic advantage in Spain (47). In our study, the evolocumab-based regimen still demonstrated a favorable economic advantage. In the base-case analysis, inclisiran had an ICER of USD 31,730.14/QALY and was less economically favorable than alirocumab and evolocumab. In a separate scenario analysis incorporating a 72.1% reduction in the acquisition price of inclisiran, its ICER decreased substantially to USD 9,686.31/QALY. This finding suggests that the economic value of inclisiran is highly sensitive to its acquisition price. However, because this estimate was derived from a price-reduction scenario and because direct cardiovascular outcome data for inclisiran remain limited, the result should be interpreted cautiously and should not be considered equivalent to the base-case finding. To date, no studies have assessed the cost-effectiveness of inclisiran at this lower price point. It should be noted, however, that large-scale RCTs with cardiovascular mortality and all-cause mortality as primary endpoints for inclisiran are still lacking. Consequently, its clinical benefits are primarily inferred from the degree of LDL-C reduction, which was translated into cardiovascular risk reduction through established LDL-C–cardiovascular outcome relationships in the economic model. This approach introduces substantial uncertainty into the estimated long-term treatment effects and consequently into the cost-effectiveness results. We acknowledge that the background lipid-lowering regimens differed across the included trials. Although a standardized atorvastatin plus ezetimibe regimen was adopted in the economic model to reflect routine clinical practice in China, this simplification may not fully capture the heterogeneity of background therapies and therefore represents a source of uncertainty in the estimated ICERs. This assumption is commonly adopted in lipid-lowering economic evaluations but should be interpreted with caution in the absence of direct cardiovascular outcomes evidence (19, 20). Moreover, the potential advantages of inclisiran related to its low dosing frequency and improved adherence have not yet been fully captured in long-term real-world data. With the release of results from ongoing cardiovascular outcome trials and further price reductions driven by NDPN policies, the cost-effectiveness profile of inclisiran may improve substantially. Accordingly, the estimated long-term cardiovascular benefits and economic value of inclisiran presented in this study should be interpreted as model-based projections rather than evidence equivalent to direct randomized cardiovascular outcome trials.

This study has some limitations. First, the clinical efficacy parameters were mainly obtained from meta-analyses assessing the efficacy and safety of PCSK9 inhibitors. Although the use of TSA and sensitivity analysis enhanced the robustness of the findings, heterogeneity among studies in terms of patient characteristics, follow-up duration, and outcome definitions may still have influenced the effect estimates. In addition, indirect comparisons in the network meta-analysis may be affected by limitations in transitivity and the absence of closed-loop evidence for some comparisons. Second, the incidence of certain cardiovascular outcomes, such as stroke and cardiovascular mortality, was relatively low, and direct comparative evidence was limited, leading to some uncertainty in the corresponding effect estimates. Finally, although this study was conducted in accordance with the PRISMA statement, the review protocol was not prospectively registered. Regarding the economic evaluation, many parameters in the Markov model—including long-term transition probabilities and health utility values—were sourced from previously published studies and may not fully reflect real-world clinical practice in Chinese patients with ASCVD. Moreover, the model used pooled efficacy estimates from a broadly defined ASCVD population, which may not capture differences across clinical subgroups, leading to potential uncertainty in subgroup-specific ICER estimates. Finally, the results may have limited generalizability to other healthcare systems due to differences in pricing structures and reimbursement policies.

## 5 Conclusion

For patients with ASCVD in the Chinese healthcare setting, evolocumab demonstrated the most favorable overall balance of lipid-lowering efficacy, cardiovascular benefits, and cost-effectiveness in the base-case analysis, while alirocumab represented another cost-effective treatment option. Inclisiran was less economically favorable under the base-case assumptions. Under the scenario incorporating a 72.1% reduction in the acquisition price of inclisiran, inclisiran emerged as a potentially highly cost-effective option within the Chinese healthcare system.

## Data Availability

The original contributions presented in the study are included in the article/Supplementary Material, further inquiries can be directed to the corresponding author.

## Footnotes

^1^ https://www.nhc.gov.cn
