# Supplementary material for: PCSK9 inhibitors for lipid-lowering treatment in patients with atherosclerotic cardiovascular disease: a health technology assessment
Source: Front Cardiovasc Med. 2026 Aug 7;13:1853767. doi: 10.3389/fcvm.2026.1853767 (PMC13493235; doi:10.3389/fcvm.2026.1853767)
Supplement: Supplementary file 1 [file Datasheet1.docx]

**Supplemental Material**

**Contents**

Appendix 1 Search strategy of electronic databases

Appendix 2 Details of the statistical analysis of meta-analysis

Appendix 3 Details of model inputs of cost-effectiveness analysis

Appendix 4 PRISMA NMA Checklist (Table S1)

Appendix 5 CHEERS 2022 Checklist (Table S2)

Appendix 6 Supplementary charts

**Appendix 1**

Search strategy of electronic databases (PubMed as sample)

Cardiovascular Diseases	5,036,392 articles 20260130

#1((brain*) OR (cerebral)) OR (lacunar)

#2infarct* AND #1

#3((Cardiovascular Diseases[MeSH Terms]) OR (cardio* OR cardia* OR heart* OR coronary* OR angina* OR ventric* OR myocard* OR pericard* OR isch?em* OR emboli* OR arrhythmi* OR tachycardi* OR endocardi* OR stroke OR strokes OR cerebrovasc* OR "cerebral vascular" OR apoplexy OR arteriosclerosis OR cholesterol[MeSH Terms])) OR (#2)

Drug	548534 articles 20260130

Monoclonal antibodies

#4((((((((((((Antibodies, Monoclonal[MeSH Terms]) OR (Antibodies,Monoclonal, Humanized[MeSH Terms])) OR (Antibodies, Monoclonal[Title/Abstract])) OR (Antibodies,Monoclonal, Humanized[Title/Abstract])) OR (monoclonal antibody[Title/Abstract])) OR (Antibody, Monoclonal[Title/Abstract])) OR (Monoclonal Antibodies[Title/Abstract])) OR (Antibodies, Humanized[Title/Abstract])) OR (Humanized Antibodies[Title/Abstract])) OR (antibodies, monoclonal, murine derived[Title/Abstract])) OR (antibodies, monoclonal, murine-derived[Title/Abstract])) OR (clonal antibody[Title/Abstract])) OR (hybridoma antibody[Title/Abstract])

siRNA

#5((((((((((((((((((((((((((((((RNA, Small Interfering[MeSH Terms]) OR (Interfering RNA, Small[Title/Abstract])) OR (siRNA[Title/Abstract])) OR (Small Interfering RNA[Title/Abstract])) OR (Short Interfering RNA[Title/Abstract])) OR (Interfering RNA, Short[Title/Abstract])) OR (RNA, Short Interfering[Title/Abstract])) OR (Scan RNA[Title/Abstract])) OR (RNA, Scan[Title/Abstract])) OR (scnRNA[Title/Abstract])) OR (Small Scan RNA[Title/Abstract])) OR (RNA, Small Scan[Title/Abstract])) OR (Scan RNA, Small[Title/Abstract])) OR (Repeat-Associated siRNA[Title/Abstract])) OR (Repeat Associated siRNA[Title/Abstract])) OR (siRNA, Repeat-Associated[Title/Abstract])) OR (siRNA, Repeat Associated[Title/Abstract])) OR (Trans-Acting siRNA[Title/Abstract])) OR (Trans Acting siRNA[Title/Abstract])) OR (siRNA, Trans-Acting[Title/Abstract])) OR (siRNA, Trans Acting[Title/Abstract])) OR (tasiRNA[Title/Abstract])) OR (Short Hairpin RNA[Title/Abstract])) OR (Hairpin RNA, Short[Title/Abstract])) OR (RNA, Short Hairpin[Title/Abstract])) OR (shRNA[Title/Abstract])) OR (Small Hairpin RNA[Title/Abstract])) OR (Hairpin RNA, Small[Title/Abstract])) OR (RNA, Small Hairpin[Title/Abstract])) OR (siRNAs[Title/Abstract])) OR (small interfering ribonucleic acid[Title/Abstract])

evolocumab

#6(((((((evolocumab[Supplementary Concept]) OR (evolocumab[Title/Abstract])) OR (repatha[Title/Abstract])) OR (AMG-145[Title/Abstract])) OR (AMG 145[Title/Abstract])) OR (amg145[Title/Abstract])) OR (sal 003[Title/Abstract])) OR (sal003[Title/Abstract])

alirocumab

#7((((((((((alirocumab[Supplementary Concept]) OR (SAR236553[Title/Abstract])) OR (SAR-236553[Title/Abstract])) OR (praluent[Title/Abstract])) OR (REGN-727[Title/Abstract])) OR (REGN727[Title/Abstract])) OR (REGN727 monoclonal antibody[Title/Abstract])) OR (monoclonal antibody REGN727[Title/Abstract])) OR (regn 727[Title/Abstract])) OR (sar 236553[Title/Abstract])) OR (alirocumab[Title/Abstract])

other

#8((((((((tafolecimab[Title/Abstract]) OR (recaticimab[Title/Abstract])) OR (ebronucimab[Title/Abstract])) OR (ak 102[Title/Abstract])) OR (ak102[Title/Abstract])) OR (Ongericimab[Title/Abstract])) OR (JS 002[Title/Abstract])) OR (JS-002[Title/Abstract])) OR (JS002[Title/Abstract])

inclisiran

#9((((((((((ALN-PCS[Supplementary Concept]) OR (ALN-PCS[Title/Abstract])) OR (inclisiran[Title/Abstract])) OR (ALN-PCSsc[Title/Abstract])) OR (leqvio[Title/Abstract])) OR (aln 60212[Title/Abstract])) OR (aln pcssc[Title/Abstract])) OR (aln60212[Title/Abstract])) OR (inclisiran sodium[Title/Abstract])) OR (kjx 839[Title/Abstract])) OR (kjx839[Title/Abstract])

PCSK9 Inhibitor

#10((((((PCSK9 Inhibitors[MeSH Terms]) OR (PCSK9 Inhibitors[Title/Abstract])) OR (PCSK9 Inhibitors, Cardiovascular[Title/Abstract])) OR (Cardiovascular PCSK9 Inhibitors[Title/Abstract])) OR (Inhibitors, Cardiovascular PCSK9[Title/Abstract])) OR (PCSK9 Inhibitors Cardiovascular[Title/Abstract])) OR (Cardiovascular, PCSK9 Inhibitors[Title/Abstract])

#11((((((#4) OR (#5)) OR (#6)) OR (#7)) OR (#8)) OR (#9)) OR (#10)

PCSK9	7417 articles 20260130

#12(((((((((((((((((((((proprotein convertase 9[MeSH Terms]) OR (proprotein convertase 9[Title/Abstract])) OR (PCSK9 protein, human[Supplementary Concept])) OR (PCSK9 protein, human[Title/Abstract])) OR (Convertase 9, Proprotein[Title/Abstract])) OR (Proprotein Convertase, Subtilisin-Kexin Type 9[Title/Abstract])) OR (Proprotein Convertase, Subtilisin Kexin Type 9[Title/Abstract])) OR (Neural Apoptosis-Regulated Convertase 1[Title/Abstract])) OR (Neural Apoptosis Regulated Convertase 1[Title/Abstract])) OR (NARC-1 Protein[Title/Abstract])) OR (NARC 1 Protein[Title/Abstract])) OR (neural apoptosis-regulated convertase 1, human[Title/Abstract])) OR (NARC-1 protein, human[Title/Abstract])) OR (NARC1 protein, human[Title/Abstract])) OR (proprotein convertase subtilisin kexin type 9, human[Title/Abstract])) OR (Pro-protein convertase subtilisin-kexin type 9, human[Title/Abstract])) OR (proprotein convertase subtilisin kexin 9, human[Title/Abstract])) OR (PCSK9 protein[Title/Abstract])) OR (proprotein convertase subtilisin/kexin type 9[Title/Abstract])) OR (protein PCSK9[Title/Abstract])) OR (PCSK9[Title/Abstract])) OR (subtilisin/kexin like protease PC9[Title/Abstract])

RCTs	Randomized controlled trials	4313145 articles 20260130

#13(((((((((((((Randomized Controlled Trials as Topic[MeSH Terms]) OR (Randomized Controlled Trials as Topic[Title/Abstract])) OR (Randomized Controlled Trial[Publication Type])) OR (Randomized Controlled Trial[Title/Abstract])) OR (Randomized controlled trials[MeSH Terms])) OR (Randomized controlled trials[Title/Abstract])) OR (Controlled Clinical trial[Publication Type])) OR (random*[Title/Abstract])) OR (randomized[Title/Abstract])) OR (randomly[Title/Abstract])) OR (groups[Title/Abstract])) OR (controlled trial, randomized[Title/Abstract])) OR (randomised controlled study[Title/Abstract])) OR (trial, randomized controlled[Title/Abstract])

#14(((#3) AND (#11)) AND (#12)) AND (#13)	1114 articles 20260130

#18Randomized controlled trials[MeSH Terms]

#19Randomized controlled trials[Publication Type]

#20Randomized Controlled trials as topic [MeSH Terms]

#21Controlled Clinical trial [Publication Type]

#22random*[Text Word]

#23randomized [Title/Abstract]

#24randomly [Title/Abstract]

#25groups [Title/Abstract]

#26#18OR#19OR#20OR#21OR#22OR#23OR#24OR#25

**Appendix 2**

For each study, six domains of bias were evaluated: (1) selection bias, including random sequence generation and allocation concealment; (2) detection bias, including blinding of outcome assessment; (3) performance bias, including blinding of participants and personnel; (4) reporting bias, including selective outcome reporting; (5) attrition bias, including incomplete outcome data; and (6) other potential sources of bias.

**Details of the statistical analysis of meta-analysis**

First, pairwise meta-analyses were performed using either fixed-effects or random-effects models, as appropriate. Effect estimates for primary and secondary outcomes were expressed as relative risks (RRs) or mean differences (MDs) with corresponding 95% confidence intervals (CIs). A two-sided P value < 0.05 was considered statistically significant. Statistical heterogeneity was assessed using the I² statistic, with I² > 50% indicating substantial heterogeneity.The methodological quality of each eligible trial was assessed according to the Cochrane Handbook, and studies were categorized as having a low, unclear, or high risk of bias. Publication bias was evaluated using funnel plots, and subgroup analyses were conducted to examine the effects of different PCSK9 inhibitors on study outcomes. The impact of population heterogeneity on outcomes was explored by including only high-quality literature and excluding high-risk patients (such as those with acute coronary syndrome, patients in the acute phase undergoing PCI, and patients who had experienced an acute ischemic stroke within the preceding 24 hours). In the included PCSK9 inhibitor trials, the intensity of background statin therapy was defined as the statin ± ezetimibe regimen with the largest number of participants. When zero events occurred in a single study arm, a continuity correction of 0.5 was applied to all comparison groups, and the Haldane method was used to handle zero-event data to ensure the stability of effect estimates^[1]^.Secondly, TSA is a statistical approach used to estimate the required information size for a meta-analysis. It is designed to control the risk of random errors arising from repeated significance testing and sparse data in cumulative meta-analyses, thereby assessing the reliability and robustness of the study conclusions^[2]^. If the cumulative Z-curve enters the futility area or crosses the trial sequential monitoring boundary, sufficient evidence is considered to have been achieved, and further trials may be unnecessary. If the Z-curve crosses only the conventional significance boundary but not the trial sequential monitoring boundary, the results may be subject to an increased risk of type I error. If the Z-curve does not cross any boundary and fails to reach the required information size, the available evidence is considered insufficient to draw definitive conclusions.

Third, NMA was conducted using Stata software (version 16.0) to compare differences in efficacy and safety among the various PCSK9 inhibitors. The PCSK9 inhibitors were ranked by calculating the surface under the cumulative ranking curve (SUCRA) probabilities for each outcome.

**Appendix 3**

Details of model inputs of cost-effectiveness analysis

**Patient population**

This study simulated the ASCVD population in clinical practice using real-world data to capture representative characteristics of patients with ASCVD. Baseline demographic and clinical characteristics were derived from the included RCTs and real-world data, with detailed information presented in **Table 2**.

**Intervention effects**

This approach, based on existing literature on the known association between LDL-C reduction and changes in cardiovascular outcome risk, is typically used in situations where direct treatment efficacy data are lacking. Linking LDL-C reduction with reduced cardiovascular risk (Cholesterol Treatment Trials Collaboration Group et al., 2010) has been widely applied in previous cost-effectiveness studies of PCSK9 inhibitors^[3,4]^. The effect of PCSK9 inhibitors on LDL-C reduction in addition to standard statin therapy is presented in our meta-analysis, showing the extent of LDL-C reduction caused by PCSK9 inhibitors (**Table S5**). Previous studies have shown that a 1 mmol/L reduction in LDL-C is associated with approximately a 15%–20% reduction in cardiovascular mortality risk and approximately a 10% reduction in all-cause mortality risk. Post-treatment event rates were estimated using Equation 1 and used as intervention effect parameters in the economic model.

$$r_{tx}=r_{0}\times{RR}^{\Delta LDLc}$$

where 𝑟_𝑡𝑥_ is the post-treatment event rate, 𝑟_0_ is the baseline event rate, 𝑅𝑅 is the rate ratio per 1.0 mmol/L LDL-C reduction, and Δ𝐿𝐷𝐿𝑐 is the absolute LDL-C reduction (mmol/L).

**Cost**

From the perspective of the Chinese healthcare system, this study considered only direct medical costs, including drug costs, hospitalization costs related to cardiovascular events, and follow-up management costs. Drug dosages were determined based on the average medication consumption of the study population, and unit drug prices were obtained from current Chinese medical insurance or hospital procurement prices. Other medical cost parameters were sourced from published studies (**Table 1**).

A Markov model was applied under the assumption of a 20% reduction in cardiovascular mortality for each 1 mmol/L decrease in LDL-C, and the corresponding ICERs are reported in Table 3. In this scenario, ICERs for all three PCSK9 inhibitors were reduced, although the ICER for inclisiran remained within the range of 1–3×GDP. Probabilistic sensitivity analysis showed that the CEAC results (Figure 6c) followed a pattern similar to the base-case analysis. The incremental cost-effectiveness scatter plots indicated that, at a WTP of 13,410 USD/QALY, both alirocumab and evolocumab were cost-effective with a probability of 100%, whereas inclisiran was not. When the WTP threshold increased to 40,230 USD/QALY, inclisiran became cost-effective (Supplementary Figure 12).

The model, incorporating a 72.1% reduction in inclisiran price, was evaluated under scenarios assuming 15% and 20% decreases in cardiovascular mortality for each 1 mmol/L reduction in LDL-C. The base-case analysis showed that, under baseline assumptions, the ICER for inclisiran decreased to 9686.31 USD/QALY, while under the scenario assuming a 20% reduction in cardiovascular mortality per 1 mmol/L LDL-C decrease, the ICER further declined to 8444.6 USD/QALY. In both scenarios, the ICER of inclisiran was lower than those of evolocumab and alirocumab under the same conditions.

In the baseline PSA, the CEAC results showed that at a WTP threshold of 13,410 USD/QALY, inclisiran was the most cost-effective treatment option, with a probability of 96.6%, compared with 2.9% for evolocumab and 0.5% for alirocumab (Figure 6b). When the WTP increased to 40,230 USD/QALY, all three PCSK9 inhibitors were superior to SOC; inclisiran remained the most cost-effective option with a probability of 48.4%, followed by evolocumab (34.8%) and alirocumab (16.8%) (Figure 6d). The incremental cost-effectiveness scatter plot indicated that, at a WTP of USD 13,410/QALY, inclisiran had a 100% probability of being cost-effective compared with SOC (Figure 7d). The PSA results under the assumption of a 20% reduction in cardiovascular mortality per 1 mmol/L decrease in LDL-C are shown in Figure 6d and Figure S11.

According to the Markov model analysis, when the price of inclisiran is USD 1,398.88 per vial, evolocumab is the most cost-effective treatment option. However, when the price of inclisiran is reduced by 72.1% to USD 390.76, its cost-effectiveness improves significantly, making it the most cost-effective option under the WTP threshold.

**Appendix 4**

PRISMA NMA Checklist (Table S1)

Table S1PRISMA 2020 checklist

| **Section and Topic** | **Item #** | **Checklist item** | **Location where item is reported** |
| --- | --- | --- | --- |
| **TITLE** | | |  |
| Title | 1 | Identify the report as a systematic review. | Title |
| **ABSTRACT** | | |  |
| Abstract | 2 | See the PRISMA 2020 for Abstracts checklist. | Abstract |
| **INTRODUCTION** | | |  |
| Rationale | 3 | Describe the rationale for the review in the context of existing knowledge. | Introduction |
| Objectives | 4 | Provide an explicit statement of the objective(s) or question(s) the review addresses. | Introduction |
| **METHODS** | | |  |
| Eligibility criteria | 5 | Specify the inclusion and exclusion criteria for the review and how studies were grouped for the syntheses. | Methods |
| Information sources | 6 | Specify all databases, registers, websites, organisations, reference lists and other sources searched or consulted to identify studies. Specify the date when each source was last searched or consulted. | Figure 1 |
| Search strategy | 7 | Present the full search strategies for all databases, registers and websites, including any filters and limits used. | Supplementary Materials Appendix 1 |
| Selection process | 8 | Specify the methods used to decide whether a study met the inclusion criteria of the review, including how many reviewers screened each record and each report retrieved, whether they worked independently, and if applicable, details of automation tools used in the process. | Methods |
| Data collection process | 9 | Specify the methods used to collect data from reports, including how many reviewers collected data from each report, whether they worked independently, any processes for obtaining or confirming data from study investigators, and if applicable, details of automation tools used in the process. | Methods |
| Data items | 10a | List and define all outcomes for which data were sought. Specify whether all results that were compatible with each outcome domain in each study were sought (e.g. for all measures, time points, analyses), and if not, the methods used to decide which results to collect. | Methods |
|  | 10b | List and define all other variables for which data were sought (e.g. participant and intervention characteristics, funding sources). Describe any assumptions made about any missing or unclear information. | Table 1, Supplementary Table 3-4 |
| Study risk of bias assessment | 11 | Specify the methods used to assess risk of bias in the included studies, including details of the tool(s) used, how many reviewers assessed each study and whether they worked independently, and if applicable, details of automation tools used in the process. | Methods |
| Effect measures | 12 | Specify for each outcome the effect measure(s) (e.g. risk ratio, mean difference) used in the synthesis or presentation of results. | Appendix 2 |
| Synthesis methods | 13a | Describe the processes used to decide which studies were eligible for each synthesis (e.g. tabulating the study intervention characteristics and comparing against the planned groups for each synthesis (item #5)). | Table 1 |
|  | 13b | Describe any methods required to prepare the data for presentation or synthesis, such as handling of missing summary statistics, or data conversions. | Appendix 2 |
|  | 13c | Describe any methods used to tabulate or visually display results of individual studies and syntheses. | Table 1 |
|  | 13d | Describe any methods used to synthesize results and provide a rationale for the choice(s). If meta-analysis was performed, describe the model(s), method(s) to identify the presence and extent of statistical heterogeneity, and software package(s) used. | Appendix 2 |
|  | 13e | Describe any methods used to explore possible causes of heterogeneity among study results (e.g. subgroup analysis, meta-regression). | Appendix 2 |
|  | 13f | Describe any sensitivity analyses conducted to assess robustness of the synthesized results. | Appendix 2 |
| Reporting bias assessment | 14 | Describe any methods used to assess risk of bias due to missing results in a synthesis (arising from reporting biases). | Appendix 2 |
| Certainty assessment | 15 | Describe any methods used to assess certainty (or confidence) in the body of evidence for an outcome. | Appendix 2 |
| **RESULTS** | | |  |
| Study selection | 16a | Describe the results of the search and selection process, from the number of records identified in the search to the number of studies included in the review, ideally using a flow diagram. | Figure 1 |
|  | 16b | Cite studies that might appear to meet the inclusion criteria, but which were excluded, and explain why they were excluded. | NA |
| Study characteristics | 17 | Cite each included study and present its characteristics. | Results |
| Risk of bias in studies | 18 | Present assessments of risk of bias for each included study. | Figure S2-S3 |
| Results of individual studies | 19 | For all outcomes, present, for each study: (a) summary statistics for each group (where appropriate) and (b) an effect estimate and its precision (e.g. confidence/credible interval), ideally using structured tables or plots. | Table 1 |
| Results of syntheses | 20a | For each synthesis, briefly summarise the characteristics and risk of bias among contributing studies. | Figure S2-S3 |
|  | 20b | Present results of all statistical syntheses conducted. If meta-analysis was done, present for each the summary estimate and its precision (e.g. confidence/credible interval) and measures of statistical heterogeneity. If comparing groups, describe the direction of the effect. | Results |
|  | 20c | Present results of all investigations of possible causes of heterogeneity among study results. | Results |
|  | 20d | Present results of all sensitivity analyses conducted to assess the robustness of the synthesized results. | Results |
| Reporting biases | 21 | Present assessments of risk of bias due to missing results (arising from reporting biases) for each synthesis assessed. | Figure S2-S11 |
| Certainty of evidence | 22 | Present assessments of certainty (or confidence) in the body of evidence for each outcome assessed. | Figure S2-S11 |
| **DISCUSSION** | | |  |
| Discussion | 23a | Provide a general interpretation of the results in the context of other evidence. | Discussion |
|  | 23b | Discuss any limitations of the evidence included in the review. | Discussion |
|  | 23c | Discuss any limitations of the review processes used. | Discussion |
|  | 23d | Discuss implications of the results for practice, policy, and future research. | Discussion |
| **OTHER INFORMATION** | | |  |
| Registration and protocol | 24a | Provide registration information for the review, including register name and registration number, or state that the review was not registered. | NA |
|  | 24b | Indicate where the review protocol can be accessed, or state that a protocol was not prepared. | NA |
|  | 24c | Describe and explain any amendments to information provided at registration or in the protocol. | NA |
| Support | 25 | Describe sources of financial or non-financial support for the review, and the role of the funders or sponsors in the review. | Funding |
| Competing interests | 26 | Declare any competing interests of review authors. | Competing interests |
| Availability of data, code and other materials | 27 | Report which of the following are publicly available and where they can be found: template data collection forms; data extracted from included studies; data used for all analyses; analytic code; any other materials used in the review. | Availability of data |

*From:* Page MJ, McKenzie JE, Bossuyt PM, Boutron I, Hoffmann TC, Mulrow CD, et al. The PRISMA 2020 statement: an updated guideline for reporting systematic reviews. BMJ 2021;372:n71. doi: 10.1136/bmj.n71. This work is licensed under CC BY 4.0. To view a copy of this license, visit <https://creativecommons.org/licenses/by/4.0/>

**Appendix 5**

Table S2 CHEERS 2022 Checklist

|  | **Item** | **Guidance for Reporting** | **Reported in section** |
| --- | --- | --- | --- |
| **TITLE** | | |  |
| Title | 1 | Identify the study as an economic evaluation and specify the interventions being compared. | Title |
| **ABSTRACT** | | |  |
| Abstract | 2 | Provide a structured summary that highlights context, key methods, results and alternative analyses. | Abstract |
| **INTRODUCTION** | | |  |
| Background and objectives | 3 | Give the context for the study, the study question and its practical relevance for decision making in policy or practice. | Introduction |
| **METHODS** | | |  |
| Health economic  analysis plan | 4 | Indicate whether a health economic analysis plan was developed and  where available. | NA |
| Study population | 5 | Describe characteristics of the study population (such as age range, demographics, socioeconomic, or clinical characteristics). | Methods |
| Setting and location | 6 | Provide relevant contextual information that may influence findings. | Appendix 3 |
| Comparators | 7 | Describe the interventions or strategies being compared and why chosen. | Methods |
| Perspective | 8 | State the perspective(s) adopted by the study and why chosen. | Methods |
| Time horizon | 9 | State the time horizon for the study and why appropriate. | Methods |
| Discount rate | 10 | Report the discount rate(s) and reason chosen. | Methods |
| Selection of outcomes | 11 | Describe what outcomes were used as the measure(s) of benefit(s) and harm(s). | Methods |
| Measurement of outcomes | 12 | Describe how outcomes used to capture benefit(s) and harm(s) were measured. | Table S3 |
| Valuation of outcomes | 13 | Describe the population and methods used to measure and value outcomes. | Methods |
| Measurement and valuation of resources  and costs | 14 | Describe how costs were valued. | Methods |
| Currency, price date, and conversion | 15 | Report the dates of the estimated resource quantities and unit costs, plus the currency and year of conversion. | Methods |
| Rationale and  description of model | 16 | If modelling is used, describe in detail and why used. Report if the model  is publicly available and where it can be accessed. | Methods |
| Analytics and assumptions | 17 | Describe any methods for analysing or statistically transforming data, any extrapolation methods, and approaches for validating any model used. | Methods |
| Characterizing heterogeneity | 18 | Describe any methods used for estimating how the results of the study vary for sub-groups. | Methods |
| Characterizing  distributional effects | 19 | Describe how impacts are distributed across different individuals  or adjustments made to reflect priority populations. | Appendix 3 |
| Characterizing uncertainty | 20 | Describe methods to characterize any sources of uncertainty in the analysis. | Methods |
| Approach to engagement with patients and others affected by the study | 21 | Describe any approaches to engage patients or service recipients, the general public, communities, or stakeholders (e.g., clinicians or payers) in the design of the study. | Methods |
| **RESULTS** | | |  |
| Study parameters | 22 | Report all analytic inputs (e.g., values, ranges, references) including uncertainty or distributional assumptions. | Table S3 |
| Summary of main results | 23 | Report the mean values for the main categories of costs and outcomes of interest and summarise them in the most appropriate overall measure. | Table 2 |
| Effect of uncertainty | 24 | Describe how uncertainty about analytic judgments, inputs, or projections  affect findings. Report the effect of choice of discount rate and time horizon, if applicable. | Figure 5-6 |
| Effect of engagement with patients and others affected by the study | 25 | Report on any difference patient/service recipient, general public, community, or stakeholder involvement made to the approach or findings of the study | Figure 5-6 |
| **DISCUSSION** | | |  |
| Study findings, limitations, generalizability, and current knowledge | 26 | Report key findings, limitations, ethical or equity considerations not captured, and how these could impact patients, policy, or practice. | Discussion |
| **OTHER RELEVANT INFORMATION** | | | |
| Source of funding | 27 | Describe how the study was funded and any role of the funder in the identification, design, conduct, and reporting of the analysis | Funding |
| Conflicts of interest | 28 | Report authors conflicts of interest according to journal or  International Committee of Medical Journal Editors requirements. | Conflicts of interest |

Husereau D, Drummond M, Augustovski F, de Bekker-Grob E, Briggs AH, Carswell C, Caulley L, Chaiyakunapruk N, Greenberg D, Loder E, Mauskopf J, Mullins CD, Petrou S, Pwu RF, Staniszewska S; CHEERS 2022 ISPOR Good Research Practices Task Force. Consolidated Health Economic Evaluation Reporting Standards 2022 (CHEERS 2022) Statement: Updated Reporting Guidance for Health Economic Evaluations. BMJ. 2022;376:e067975.

The checklist is Open Access distributed in accordance with the terms of the Creative Commons Attribution (CC BY 4.0) license, which permits others to distribute, remix, adapt and build upon this work, for commercial use, provided the original work is properly cited. See: [http://creativecommons.org/licenses/by/4.0/.](http://creativecommons.org/licenses/by/4.0/)

**Appendix 6**

Figure S1 Markov state transition model for ASCVD patients

Figure S2 Risk of bias summary: review authors' judgements about each risk of bias item for each included study.

Figure S3 Risk of bias graph: review authors' judgements about each risk of bias item presented as percentages across all included studies

Figure S4 Funnel plot for publication bias assessment of LDL-C

Figure S5 Effects of PCSK9 inhibitors compared to placebo on SAE

Figure A shows the forest plot of subgroup analysis from a paired meta-analysis of PCSK9 inhibitors; Figure B shows the sequential trial analysis, using a mean difference model for sequential trial analysis. The standard deviation was derived from the pooled standard deviation of LDL-C in the control groups of the included studies, with a two-sided α=0.05 and a power of 80%.

CI, confidence interval; PCSK9, proprotein convertase subtilisin/kexin type 9.

Figure S6 Funnel plot for publication bias assessment of SAE

Figure S7 Forest plot for pairwise meta-analysis of Lp(a)

Figure S8 Forest plot for pairwise meta-analysis of apoB

Figure S9 Forest plot for pairwise meta-analysis of NMI

Figure S10 Forest plot for pairwise meta-analysis of stroke

Figure S11 Forest plot for pairwise meta-analysis of LDL-C (Only high-quality literature was included)

Figure S12 Forest plot for pairwise meta-analysis of LDL-C (Exclude high-risk subgroups)

Figure S13 Forest plot for pairwise meta-analysis of SAE (Only high-quality literature was included)

Figure S14 Forest plot for pairwise meta-analysis of SAE (Exclude high-risk subgroups)

Figure S15 Forest plot for pairwise meta-analysis of NMI (Only high-quality literature was included)

Figure S16 Forest plot for pairwise meta-analysis of NMI (Only high-quality literature was included)

Figure S17 Forest plot for pairwise meta-analysis of Stroke (Only high-quality literature was included)

Figure S18 Forest plot for pairwise meta-analysis of Stroke (Only high-quality literature was included)

Figure S19 Funnel plots for publication bias assessment.

Figure S20 Scatter plot of incremental cost-effectiveness of three PCSK9 inhibitors compared to SOC when LDL-C decreases by 1 mmol/L, resulting in a 20% reduction in cardiovascular mortality.

**Appendix 7**

Table S3 Key inputs in the markov model.

| Parameter | Value | Range | Distribution | | Source |
| --- | --- | --- | --- | --- | --- |
| Event rate per 100 patient-years | | | | | |
| Nonfatal MI | 1.7 | Age dependent | NA | | [23] |
| Nonfatal stroke | 0.9 | Age dependent | NA | | [23] |
| Cardiovascular-related death | 1.9 | Age dependent | NA | | [23] |
| All-cause mortality | 5.1 | Age dependent | NA | | [21] |
| Health utility value | | | | | |
| ASCVD | 0.824 | 0.800–0.848 | Log normal | | [20] |
| Nonfatal MI (year 1) | 0.672 | 0.625–0.719 | Log normal | | [20] |
| Nonfatal MI (beyond 1y) | 0.824 | 0.800–0.848 | Log normal | | [20] |
| Nonfatal stroke (year 1) | 0.327 | 0.264–0.390 | Log normal | | [20] |
| Nonfatal stroke (beyond 1y) | 0.524 | 0.472–0.576 | Log normal | | [20] |
| Cardiovascular death | 0 | - | NA | |  |
| Non-cardiovascular death | 0 | - | NA | |  |
| Annual cost of drugs, CNY | | | | | |
| Atorvastatin | 298.278 | - | NA | | Hospital purchase price (First Affiliated Hospital of Xi'an Jiaotong University) |
| Ezetimibe | 473.150 | - | NA | |  |
| Alirocumab | 7558.2 | - | NA | |  |
| Evolocumab | 7365.8 | - | NA | |  |
| Inclisiran | 19976 | - | NA | |  |
| Annual cost of the event, CNY | | | | | |
| ASCVD | 2855.15 | 118.86-5470.28 | Gamma | | [24] |
| Non-fatal MI | 28556.34 | 21417.26-35695.43 | Gamma | | [24] |
| Non-fatal stroke | 15264.07 | 11448.05-19080.09 | Gamma | | [24] |
| MI maintenance therapy | 13377.54 | 10033.15-16721.92 | Gamma | | [24] |
| Stroke maintenance therapy | 23454.42 | 17590.82-29318.03 | Gamma | | [24] |
| Death from myocardial infarction | 25177.23 | 18882.92-31471.53 | Gamma | [24] | |
| Death from stroke | 17459.61 | 13094.71-21824.51 | Gamma | [24] | |
| Other parameters | | | | | |
| Discount rate | 5 | 0-8 |  |  | |

MI, myocardial infarction; CNY, Chinese Yuan; NA, not applicable;

Table S4 Subgroup analysis of PCSK9 inhibitors in the pairwise meta-analysis of ASCVD patients

| Treatment comparisons | Number of studies | Pairwise meta-analysis | P value | Heterogeneity I^2^, % |
| --- | --- | --- | --- | --- |
| **LDL-C** |  | Mean Difference(95% CI) |  |  |
| Alirocumab vs Placebo | 6 | **-1.25(-1.51,-0.99)** | ＜0.00001 | 98 |
| Evolocumab vs Placebo | 7 | **-1.28(-1.58,-0.99)** | ＜0.00001 | 90 |
| Inclisiran vs Placebo | 1 | **-1.23(-1.37,-1.09)** | ＜0.00001 | - |
| **SAE** |  | Risk Ratio (95% CI) |  |  |
| Alirocumab vs Placebo | 6 | **0.94(0.9,0.99)** | 0.02 | 0 |
| Evolocumab vs Placebo | 6 | 1.00(0.97,1.04) | 0.81 | 0 |
| Inclisiran vs Placebo | 4 | 0.97(0.79,1.17) | 0.73 | 48 |

Table S5 PCSK9 inhibitors for ASCVD patients SUCRA ranking

| Treatment | Standard analysis | PrBest | SUCRA | SUCRA rank |
| --- | --- | --- | --- | --- |
| **LDL-C** |  |  |  |  |
| Alirocumab | **0.28 (0.21,0.37)** | 16 | 57.7 | 3 |
| **Evolocumab** | **0.31 (0.24,0.40)** | 42.4 | 75.3 | 1 |
| Inclisiran | **0.29 (0.14,0.60)** | 41.5 | 67 | 2 |
| Placebo | reference | 0.0 | 0.0 | 4 |
| **SAE** |  |  |  |  |
| **Alirocumab** | **0.93 (0.87,0.99)** | 0.4 | 17.6 | 1 |
| Evolocumab | 1.01 (0.96,1.06) | 53.1 | 80.6 | 4 |
| Inclisiran | 0.92 (0.79,1.08) | 12.8 | 27.4 | 2 |
| Placebo | reference | 33.7 | 74.4 | 3 |
| **NMI** |  |  |  |  |
| Alirocumab | **0.86 (0.77,0.96)** | 1.0 | 48.4 | 3 |
| **Evolocumab** | **0.73 (0.65,0.81)** | 69.9 | 89.7 | 1 |
| Inclisiran | 0.82 (0.52,1.30) | 29.1 | 54.7 | 2 |
| Placebo | reference | 0.0 | 7.1 | 4 |
| **Stroke** |  |  |  |  |
| Alirocumab | 0.74 (0.32,1.68) | 27.7 | 58.6 | 2 |
| **Evolocumab** | 0.55 (0.18,1.67) | 57.4 | 76.4 | 1 |
| Inclisiran | 0.94 (0.34,2.55) | 13.2 | 37.2 | 3 |
| Placebo | reference | 1.7 | 27.8 | 4 |

Table S6 Therapeutic effects of different PCSK9 inhibitors from pairwise meta-analysis

|  | Alirocumab | Evolocumab | Inclisiran |
| --- | --- | --- | --- |
| Non-fatal myocardial infarction | 0.87 (0.79,0.96) | 0.74 (0.67,0.82) | 0.81 (0.53,1.26) |
| Non-fatal stroke | 0.73 (0.58,0.93) | 0.76 (0.64,0.91) | 0.76 (0.64,0.91) |
| LDL-C | -1.25 (-1.51, -0.99) | -1.28 (-1.58, -0.99) | -1.23 (-1.37, -1.09) |
| Calculated cardiovascular mortality rate (15%) | 0.82 (0.78,0.85) | 0.81 (0.77,0.85) | 0.82 (0.8,0.84) |
| Calculated cardiovascular mortality rate (20%) | 0.76 (0.72-0.8) | 0.75 (0.7,0.8) | 0.76 (0.74,0.78) |
| Calculated all-cause mortality rate | 0.88 (0.85-0.9) | 0.87 (0.85-0.9) | 0.88 (0.87,0.89) |

Table S7 Age-specific transition probabilities in the basic analysis

| Age in events, years | Placebo, % |
| --- | --- |
| NMI | |
| 55-59 | 1.25 |
| 60-64 | 1.70 |
| 65-69 | 2.76 |
| 70-74 | 3.81 |
| 75-79 | 6.15 |
| IS | |
| 55-59 | 0.65 |
| 60-64 | 0.90 |
| 65-69 | 1.69 |
| 70-74 | 2.88 |
| 75-79 | 5.17 |
| Cardiovascular death | |
| 55-59 | 1.65 |
| 60-64 | 2.77 |
| 65-69 | 4.90 |
| 70-74 | 9.14 |
| 75-79 | 17.17 |
| Cerebrovascular death | |
| 55-59 | 0.78 |
| 60-64 | 1.33 |
| 65-69 | 2.41 |
| 70-74 | 4.59 |
| 75-79 | 8.45 |
| All-cause mortality rate | |
| 55-59 | 4.44 |
| 60-64 | 7.19 |
| 65-69 | 12.15 |
| 70-74 | 20.07 |
| 75-79 | 34.18 |

IS: ischemic stroke; MI: myocardial infarction

References

[1]	J.B. Haldane, The estimation and significance of the logarithm of a ratio of frequencies, Ann Hum Genet 20 (1956) 309–311. https://doi.org/10.1111/j.1469-1809.1955.tb01285.x.

[2]	J. Wetterslev, K. Thorlund, J. Brok, C. Gluud, Trial sequential analysis may establish when firm evidence is reached in cumulative meta-analysis, J Clin Epidemiol 61 (2008) 64–75. https://doi.org/10.1016/j.jclinepi.2007.03.013.

[3]	W. Y, L. J, Z. X, Z. Y, Y. R, Methodology and results of cost-effectiveness of LDL-C lowering with evolocumab in patients with acute myocardial infarction in China, Cost Effectiveness and Resource Allocation : C/E 21 (2023). https://doi.org/10.1186/s12962-023-00501-4.

[4]	P. Wang, L. Liang, Y. Li, Cost-effectiveness analysis of evolocumab or inclisiran in combination with statins versus statin monotherapy among patients with ASCVD in China, Appl Health Econ Health Policy 23 (2025) 855–867. https://doi.org/10.1007/s40258-025-00971-y.
